# Supplementary material for: Substantially reducing global PM2.5-related deaths under SDG3.9 requires better air pollution control and healthcare
Source: Nat Commun. 2024 Mar 28;15:2729. doi: 10.1038/s41467-024-46969-3 (PMC10978932; doi:10.1038/s41467-024-46969-3)
Supplement: Supplementary file 1 — Supplementary Information [file 41467_2024_46969_MOESM1_ESM.pdf]

Supplementary Materials

Substantially reducing global $PM_{2.5}$-related deaths under SDG3.9 requires better air pollution control and healthcare

Yue et al.

Contents

Supplementary Note 1 - Study area and data....3
Supplementary Note 2 - Estimation of deaths attributable to $PM_{2.5}$ pollution....5
Supplementary Note 3 - Projection of future $PM_{2.5}$ concentration....8
Supplementary Note 4 - Projection of future death rate of diseases. 11
Fig. S1. Definition of five regions....14
Fig. S2. Flow chart for estimating the future deaths attributable to $PM_{2.5}$ pollution. 15
Fig. S3. Exposure-response functions of Bayesian, regularized, trimmed (MR-BRT) model derived from GBD 2019 Risk Factors Collaborators (2020)....16
Fig. S4. Trends of deaths attributable to $PM_{2.5}$ pollution by model....17
Fig. S5. Trends of deaths attributable to $PM_{2.5}$ pollution by age group. 18
Fig. S6. Comparison of deaths attributable to $PM_{2.5}$ pollution between our estimates and GBD 2019 Risk Factors Collaborators (2020)....19
Fig. S7. Changes in DAPP estimated based on the lower value of the exposure-response function. 20
Fig. S8. Changes in DAPP estimated based on the upper value of the exposure-response function. 21
Fig. S9. Trends of population-weighted $PM_{2.5}$ concentration by model. 22
Fig. S10. Validation of projected $PM_{2.5}$ concentration (multi-model average)....23
Fig. S12. Comparison of multi-model averaged $PM_{2.5}$ concentration between our projection (solid line) and Turnock et al (2020) (dashed line)....25
Fig. S13. Comparison of historical socio-demographic index (SDI) between our study and GBD 2019....26
Fig. S14. Validation of the death rate projection. 27
Fig. S15. Global changes in the driving factors of DAPP. 28
Fig. S16. Region-specific changes in the driving factors of DAPP....36
Fig. S17. Histogram for historical change rate in DAPP between 2000 and 2015....37
Fig. S18. Framework of the decomposition analysis....38
Fig. S19. Region-specific effect of different driving factors on the changes in DAPP....39
Fig. S20. Country-specific effects of different driving factors on the changes in DAPP from 2015 to 2030. 40
Fig. S21. Country-specific attainment of SDG3.9 by 2030 under different combinations of scenarios as well as additional improvement in air pollution control and healthcare. 41
Fig. S22. Attainment of SDG3.9 based on the medium, lower and upper value of the exposure-response function (E-R). 42
Table S1. Comparative summary of scenario, input data and major results among previous studies....43
Table S2. Number of countries that achieved the moderate SDG3.9 by 2030 under different scenarios....44
Table S3. Details of input data for $PM_{2.5}$ concentration projection....45
References....46

Supplementary Note 1 - Study area and data.

This study was conducted on a global scale. Due to the accessibility of data, we considered only the 154 countries with a population greater than 1 million in 2015. These countries represent 99% of the global human population. Then, following the International Institute for Applied Systems Analysis (IIASA) standard [1], we aggregated these countries into 5 regions. Among them, ASIA includes Asian countries except for the Middle East, Japan, and the former Soviet Union states; OECD includes the member states of Organization for Economic Co-operation and Development and new European Union and candidates; REF includes the reforming economies of Eastern Europe and the Former Soviet Union; MAF and LAM include countries of the Middle East and Africa and countries of Latin America and the Caribbean, respectively (Fig. S1).

Historical population data was derived from the World Population Prospects 2019 (https://population.un.org/wpp/), which includes the annual country- and age-specific population from 2000 to 2019 (UN, 2019). The future projection of population and age structure was derived from the Wittgenstein Centre Human Capital Data (WIC2.0) (http://dataexplorer.wittgensteincentre.org/wcde-v2/) developed by IIASA. This dataset includes the country- and age-specific population under five Shared Socioeconomic Pathways (SSPs) for every 5 years from 2015 to 2100 worldwide [2]. The gridded population data were obtained from the History Database of the Global Environment (HYDE3.2) published by the Netherlands Environmental Assessment Agency (ftp://ftp.pbl.nl/hyde). This dataset provided the global historical (2000-2015) and future (2010-2100) gridded population under different SSPs by downscaling the national population to pixel scale, with a spatial resolution of 0.1 degrees [3].

Historical estimation of $PM_{2.5}$ concentrations were obtained from the Global Estimates of Fine Particulate Matter dataset (V4.GL.02) published by the Dalhousie University's Atmospheric Composition Analysis Group (http://fizz.phys.dal.ca/~atmos/), which includes global annual average $PM_{2.5}$ concentration from 1998-2018 with a resolution of 0.1 degrees. This dataset was generated by combining a chemical transport model, remotely sensed data, and monitoring data, which considered both coverage and accuracy [4]. The future concentration of pollutants was derived from the Coupled Model Intercomparison Project Phase 6 (CMIP6) (https://pcmdi.llnl.gov/CMIP6/), which includes monthly surface concentration of pollutants such

as SO_4, black carbon, organic aerosol, dust, and sea salt simulated by multiple models (Table S3), with a spatial resolution of about 1 degree and time span of 2015-2100 [5].

Historical death rate of diseases was accessed from the Global Burden of Disease (GBD) 2019 (http://ghdx.healthdata.org/gbd-results-tool), which includes the annual age and disease-specific death rate for 296 countries and regions worldwide from 1980 to 2019 [6].

Socioeconomic data includes fertility, GDP per capita, and average number of years of education. The fertility and mean years of education data was derived from the WIC2.0 dataset (http://dataexplorer.wittgensteincentre.org/wcde-v2/). Historical GDP per capita was sourced from publicly available World Bank [7] data and future GDP per capita data was sourced from the projections conducted by the OECD contained in the SSPs database (https://tntcat.iiasa.ac.at/SspDb/) [8].

Regarding the gridded data, we resampled all the data into a resolution of 1 degree. Regarding the country-specific statistical data, we calibrated this data for the different scenarios by calculating relative change from the base year of 2015.

Supplementary Note 2 - Estimation of deaths attributable to $PM_{2.5}$ pollution.

We estimated DAPP in the past (2000-2015) and the future (2015-2050) under 4 scenarios and 11 available CMIP6 models with the comparative risk assessment framework to estimate DAPP [9]. The comparative risk assessment framework is the most commonly used method to estimate the health impacts caused by a certain risk factor, and has been widely used in health impact assessments conducted by the Global Burden of Disease (GBD) studies and the World Health Organization (WHO) at both regional and global scale [10,11]. The key inputs of this method include population, age structure, annual average $PM_{2.5}$ concentration as well as death rate of diseases [12]. Of the driving factors used for projecting DAPP, population and age structure were derived from the existing projections in the Shared Socioeconomic Pathways (SSPs) database, which aligns with the ScenarioMIP framework [2]. Future $PM_{2.5}$ concentration and death rate of diseases were further estimated in this study. Detailed information about future $PM_{2.5}$ concentration and death rate of diseases can be found in Supplementary Note 3 and Supplementary Note 4.

Specifically, DAPP is determined by four factors, i.e., the population attributable fraction (PAF), population, death rate of diseases, and the age structure. The equation for estimating DAPP is provided below [10,13,14]:

$$DAPP = \sum_{a,d}\left(PAF_{a,d} \times POP \times Rate_{a,d} \times AgeP_a\right) \tag{2.1}$$

where $DAPP$ is the deaths attributable to $PM_{2.5}$ pollution; $PAF_{a,d}$ refers to the population attributable fraction for disease d in a population of age a; and POP refers to the total population; $Rate_{a,d}$ is the death rate of disease d for people of age a, and the $AgeP_a$ is the percentage of the total population of age a. In our model, 15 age groups were included in the equation, i.e., 25-30, 30-35…90-95, and beyond 95 years old. Six major diseases related to $PM_{2.5}$ pollution were considered in this study, including lung cancer, chronic obstructive pulmonary disease, lower respiratory infection, ischemic heart disease, stroke, and diabetes mellitus type 2. Evidence linking these diseases with exposure to ambient air pollution was judged to be consistent with a causal relationship on the basis of criteria specified for GBD risk factors, including meta-analysis, cohort study, and biologically plausible relationship[11]. We did not further consider the difference by gender to reduce the complexity of our projections. Although the death rate of diseases between

male and female is different, the sex ratio in the future is relatively constant, as will be the corresponding ratio of DAPP by gender[2].

$PAF_{a,d}$ refers to the proposition that a given disease proportion of deaths attributed to $PM_{2.5}$ pollution caused by disease d in a population with age a [15], which can be calculated as below.

$$PAF_{a,d} = \frac{RR_{a,d} - 1}{RR_{a,d}} \tag{2.2}$$

where $RR_{a,d}$ is the relative risk for the population with age a acquiring disease d. This refers to the ratio of incidence for an exposed population compared to an unexposed population. Relative risk can be quantified based on a non-linear exposure-response function. Previous works had several different options for exposure-response function [16,17]. In this study, we used the latest meta-regression – Bayesian, regularized, trimmed (MR-BRT) model exposure-response functions updated by the Global Burden of Disease (GBD) 2019 (Fig. S3). For ischemic heart disease and stroke, we used age-specific exposure-response function because the epidemiological evidence suggests that the relative risks for these diseases decline with age[12,17]. For other four diseases, the exposure-response functions are uniform for all age groups. The MR-BRT provided medium estimation as well as 95th confidence interval (Fig. S3), openly available at https://ghdx.healthdata.org/record/ihme-data/gbd-2019-burden-by-risk-1990-2019 [11]. As a result, we can derive age, disease, scenario and model specific DAPP. The trend of DAPP by model and age group can be found in Fig. S4 and Fig. S5.

We compared our results with the historical (2000-2015) DAPP estimated by the latest GBD 2019 studies. The estimates of DAPP were highly correlated, with an R^2 of 0.97 (Fig. S6). This means that our estimation of DAPP during the historical period is consistent with the widely recognized GBD results in terms of long-term spatiotemporal trends.

Finally, we also estimated the uncertainty in estimating DAPP. The driving factors and the epidemiological model both introduce uncertainty into the projection of DAPP. We present the impact of uncertainty in future $PM_{2.5}$ concentration and the death rate of diseases (Details in Supplementary Notes 3-4) on the results, calculating the possible range in DAPP based on these uncertainty intervals. Regarding the epidemiological model, uncertainty mainly comes from the choice of the exposure-response function, which reflects the relationship between $PM_{2.5}$

concentration and the risk of incidence for a specific disease. In the main text, we presented the results estimated by using medium value of the MR-BRT model as an estimate of central tendency. To validate the robustness of our findings, we obtained the upper and lower estimation of the MR-BRT model following previous studies [10,12], then again estimated the trend of DAPP (Fig. S7, Fig. S8) and the attainment of SDG3.9 (Fig. S22).

Supplementary Note 3 - Projection of future $PM_{2.5}$ concentration.

Following the established empirical algorithm from Silva et al. (2017) [18], we used data from 11 climate and earth system models in the CMIP6 database to estimate future $PM_{2.5}$ concentration, including model specific (Fig. S9) and multi-model averaged results (Fig. S15).

Specifically, 8 models in CMIP6 are available for all four scenarios in this study. In addition, one model (GFDL-CM4) was available for SSP2-4.5 and 5-8.5, and two models (BCC-ESM1 and CNRM-ESM2-1) were available for SSP3-7.0 (Table S3). Under a given scenario, these models used consistent anthropogenic and biomass-burning emissions from the same dataset[19,20], but differ in other natural emissions (e.g., dust, biogenic volatile organic compounds etc.) and aerosol scheme[21]. For example, only GISS-E2-1-G and GFDL-ESM4 have provided ammonium and nitrate mass mixing ratios. For the CNRM-ESM2-1 model, anomalously large concentrations were obtained from the sea salt mass mixing ratios. To ensure the consistency, we calculated $PM_{2.5}$ concentration offline with surface concentration of pollutants via the below equation:

$$\mathrm{PM}_{2.5} = BC + OA + SO_4 + NH_4 + 0.25 \times SS + 0.1 \times dust \tag{3.1}$$

where *BC* is black carbon, *OA* is organic aerosols, and *SS* is sea salt. The concentration of NH_4 is not specifically output by CMIP6 models so we estimated it as $NH_4 = (36 \times SO_4)/96$ assuming that NH_4 is only present as ammonium sulphate [22]. The factors 0.25 and 0.1 are intended to approximate the fractions of sea salt and dust that are in the $PM_{2.5}$ size range. As one of component of $PM_{2.5}$, nitrate was only reported by two models from CMIP6, therefore, following the treatment by Silva et al. (2013) [23] and Silva et al. (2016) [24], we did not consider it in the $PM_{2.5}$ concentration formula to avoid inconsistencies with other CMIP6 models. Some models include multiple ensembles of experiment, a mean is taken using all available members for each model. On the basis model specific $PM_{2.5}$ concentration, we calculated multi-model averaged $PM_{2.5}$ concentration as the major results.

The simulated $PM_{2.5}$ concentration is not directly comparable with the empirically estimated $PM_{2.5}$ data derived from remote sensing and monitoring stations [25]. Our simulated $PM_{2.5}$ concentrations tend to be lower than the empirically estimated $PM_{2.5}$ data and mainly reflects the long-term trends and as such, the annual variation is smaller. Considering the likely advantage in accuracy and coverage, we used the estimated $PM_{2.5}$ concentration as the baseline to project future

$PM_{2.5}$ concentration by assuming that the interannual trends of $PM_{2.5}$ concentration from the simulated and estimated data sources are consistent. Future $PM_{2.5}$ concentration was then extrapolated based on the estimated $PM_{2.5}$ concentration in the base year and the relative change in simulated $PM_{2.5}$ concentration. In addition, we used three strategies (no moving average, 3-year-moving average, and 5-year-moving average) to determine the $PM_{2.5}$ concentration at the base year to avoid the influence of extreme values in estimated data. The extrapolation equation is as follows:

$$PC_{t+n} = \frac{MC_{t+n}}{MC_t} \times EC_t \tag{3.2}$$

where *PC* represents the projected $PM_{2.5}$ concentration, *MC* and *EC* refer to the modeled and estimated $PM_{2.5}$ concentration, *t* refers to the base year, which is 2015, *n* refers to the time-span of extrapolation. For instance, the future $PM_{2.5}$ concentration in 2020 can be extrapolated using the estimated $PM_{2.5}$ concentration in 2015 with a time-span of 5 years.

We validated this projection method by extrapolating the $PM_{2.5}$ concentration in 2005, 2010 and 2015 using the same method with a time-span of 5, 10, and 15 years, and checked it against the estimated values. The results indicate that projected $PM_{2.5}$ concentration showed a good agreement with the estimated value, which means this method is suitable for projecting future $PM_{2.5}$ concentrations (Fig. S10). Among the three strategies used to determine the base year $PM_{2.5}$ concentration, we adopted the 3-year-moving average historical $PM_{2.5}$ concentration which best ensured the consistency between the estimated and extrapolated value while honoring the original information as much as possible, with the R^2 values between projected and historical $PM_{2.5}$ concentration of 0.93, 0.92 and 0.88 for extrapolating forward 5, 10, and 15 years, respectively.

In addition, we compared our results with future $PM_{2.5}$ concentrations as projected by Turnock et al. (2020) [21] based on the output of CMIP6. In summary, the global population-weighted $PM_{2.5}$ concentration between these two studies are highly consistent under most scenarios. Under the Sustainability scenario, the population-weighted $PM_{2.5}$ concentration estimated by this study is slightly lower than that from Turnock et al. (2020) [21], the difference in population-weighted $PM_{2.5}$ concentration between these two studies is around 3μg/m^3 at 2050. This provides confidence in and further confirms the reliability of our projections of $PM_{2.5}$ concentration (Fig. S12).

In this study, we used the outputs from 11 individual models to project future $PM_{2.5}$ concentration. While we used the average value of different models as the final projection of $PM_{2.5}$ concentration, we also quantified the corresponding 95% confidence interval based on the difference among the projected results from each model. By assuming that the results derived from different models comply with the Student's *T* distribution, the 95% confidence intervals of projected $PM_{2.5}$ concentration can be expressed as:

$$Con_{CI} = Con_{mean} \pm \sqrt{Var(Con)} * T_{95\%}(n-1) \tag{3.3}$$

where Con_{CI} represents the 95% confidence interval of $PM_{2.5}$ concentration, Con_{mean} and $Var(Con)$ refers to the mean value and variance of $PM_{2.5}$ concentration calculated by multiple models. $T_{95\%}(n-1)$ represents the student *T* statistic under 95% confidence interval with degrees of freedom of *n-1*, while *n* represents the number of related models.

Supplementary Note 4 - Projection of future death rate of diseases.

We further estimated future death rates of diseases based on the historical death rates of diseases and future socioeconomic development indicators with the model developed by Foreman et al. (2018) [26]. This model can reflect the long-term relationship between the variances of death rates and socioeconomic development, and further improved the projection accuracy by using the autoregressive integrated moving average (ARIMA) model [27].

Specifically, the projected death rate for a specific disease can be expressed as a function composed by a linear regression model and the projected residual:

$$\ln{(m)} = \hat{y} + \hat{\epsilon} \tag{4.1}$$

where *ln(m)* refers to the natural logarithm of disease-specific death rate, $\hat{y}$ refers to the expectation of *ln(m)* in a linear regression model, and $\hat{\epsilon}$ refers to the expectation of latent trend that cannot be explained by the regression model.

First, the disease-specific death rate and its drivers follow the linear relationship as below.

$$ln(m) \sim N(\hat{y}, \sigma) \tag{4.2}$$

$$\hat{y} = \beta_1 SDI_{<0.8} + \beta_2 SDI_{\geq 0.8} + \theta_a t + \alpha_{ca} + ln(R) \tag{4.3}$$

β_1 and β_2 represent a global coefficient of the Socio-Demographic Index (*SDI*) when the *SDI* is less than or greater than 0.8, respectively. θ_a refers to an age-specific secular trend, and t represents time. α_{ca} is the country- and age-specific intercept, and *ln(R)* represents a scalar that captures the effects of risk factors. $\hat{y}$ can be estimated according to the change in each driving factor. Because of data limitations, we assumed that the effects of risk factors are fixed in the future and hence, set *ln(R)* to equal 0 in this study.

SDI is a metric to represent socioeconomic development, which can be calculated by combining the logarithm of income per person, educational attainment, and total fertility rate under 25 years as below:

$$SDI = \sqrt[3]{Z(\log{(income)}) \times Z(education) \times Z(fertility)} \tag{4.4}$$

where *Z(x)* represents a standardization function. These three indicators can be rescaled to values between 0 and 1 by linearly rescaling from the worst to the best possible values. Then, a composite SDI ranging from 0 to 1 was computed by taking the geometric mean of the three indicators.

Second, in estimating disease-specific death rate, the *ln(m)* and $\hat{y}$ are not equal, the difference between them can be defined as ϵ, which represents the latent trend not captured by the regression model. According to the ARIMA model, the ϵ in year *t* and *t*-1 follows a linear relationship. The future ϵ can be estimated based on historical data. However, running independent ARIMA models on the ϵ of every cause, country, and age is not a robust approach and might lead to extreme forecasts. Therefore, we used a pooled model, which enabled the sharing of ARIMA parameters within regions (*r*). Hence, the final equation can be expressed as:

$$\epsilon_{cat} \sim N(\hat{\epsilon}_{cat}, \sigma) \tag{4.5}$$

$$\hat{\epsilon}_{cat} = \omega_{ra} \times \hat{\epsilon}_{ca(t-1)} \tag{4.6}$$

where $\hat{\epsilon}_{cat}$ and $\hat{\epsilon}_{cat-1}$ refer to the $\hat{\epsilon}$ in year *t* and *t-1* for country *c* and age group a. ω_{ra} is the coefficient of regression model for region *r* and age group a, which can be estimated by fitting the ϵ_{cat} and ϵ_{cat-1} over the historical period.

We further validated the projected death rate of diseases from two aspects. First, we compared the historical SDI calculated by this study and the SDI published by the GBD 2019 [6], as the SDI is the most important input for the death rate projection model. The linear regression between SDI derived from our study and GBD 2019 yielded an R^2 of 0.94 (Fig. S13), indicating that our estimation of SDI was applicable to the death rate projection model. Second, we cross-validated the death rate projection model. We projected the death rate of disease from 2005 to 2015 using the model trained by data from 1990-2015 and calculated the corresponding DAPP. Globally, the DAPP calculated based on the projected death rate of diseases was highly consistent with the reference value, with a <2% difference. At the regional scale, the DAPP based on the projected death rate of diseases was also highly aligned with the reference value, except for LAM and OECD in which DAPP was around 5% higher (Fig. S14). This validation means the above-mentioned model can support the projection of future DAPP.

In addition, we quantified the confidence intervals of the projected death rates of diseases. Because the ϵ itself represents the possible residuals in the process of linear regression model, we used ϵ's prediction error to quantify the 95% confidence intervals of the projected death rate of diseases, which can be calculated based on the commonly used linear regression method. Specifically, the prediction error of ϵ follows a Gaussian distribution with a mean of 0 and variance of $Var(e_0)$. Hence, the 95% confidence interval of *ln(m)* can be expressed as:

$$\ln(m)_{CI} = \hat{y} + \hat{\epsilon} \pm \sqrt{Var(\epsilon - \hat{\epsilon})} * T_{95\%}(n-2) \quad (4.7)$$

where $ln(m)_{CI}$ represents the 95% confidence interval of natural logarithm of disease-specific death rate. The sum of $\hat{y}$ and $\hat{\epsilon}$ refers to the projected value of *ln(m)*, and $Var(\epsilon - \hat{\epsilon})$ refers to the variation of residuals in the ARIMA model (Equation 4.6). $T_{95\%}(n-2)$ represents Student's *T* statistic under the 95% confidence interval with degrees of freedom of *n-2*, while *n* represents the number of samples in the ARIMA model.

$Var(\epsilon - \hat{\epsilon})$ can be further calculated with the following equation:

$$Var(\epsilon - \hat{\epsilon}) = \frac{\sum {e_i}^2}{n-2} \times \left[\frac{1}{n} \times \frac{(x_f - \bar{x})^2}{(x_i - \bar{x})^2}\right] \quad (4.8)$$

where $\sum {e_i}^2$ represents the residual sum of squares in ARIMA model, and x_i and $\bar{x}$ represent every independent variable and its average value. x_f represents the input value of independent variable to the ARIMA model in the process of projection.

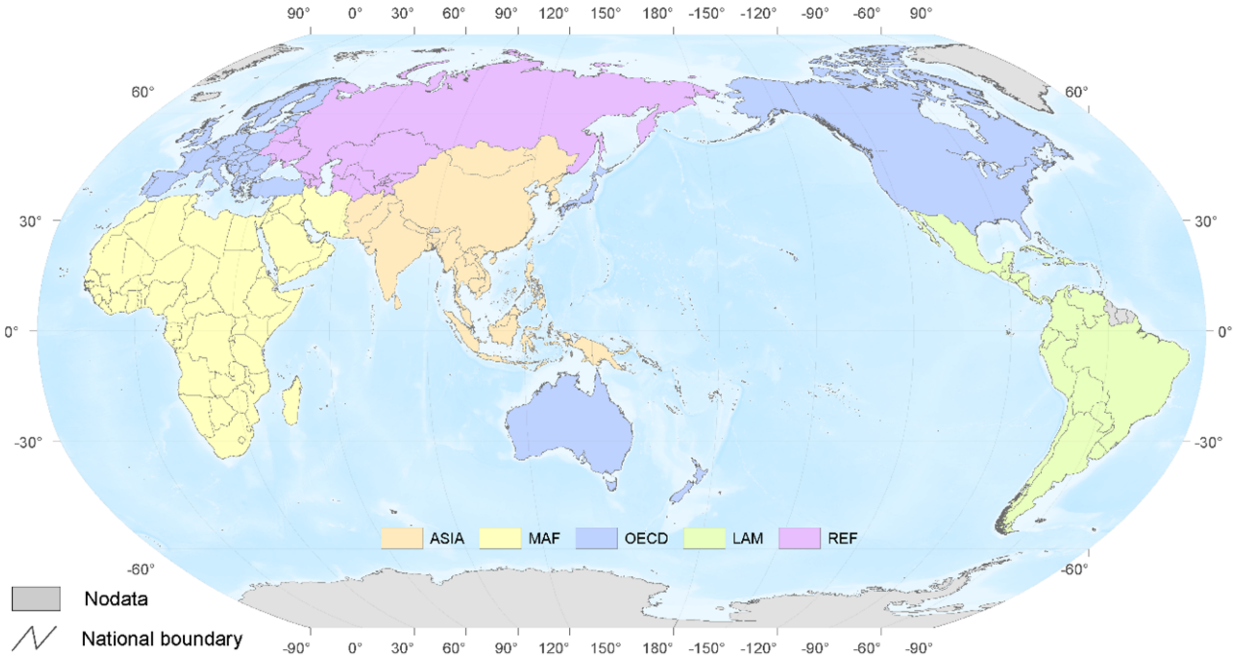

Fig. S1. Definition of five regions. OECD - member states of Organization for Economic Co-operation and Development and new European Union and candidates. REF - reforming economies of Eastern Europe and the former Soviet Union. MAF - countries of the Middle East and Africa. LAM - countries of Latin America and the Caribbean. ASIA - Asian countries (with the exception of the Middle East, Japan and Former Soviet Union states). The region definitions are from the SSPs dataset[1]. The base map is made by authors with national boundary data from the National Platform for Common Geospatial Information Services of China[28] and water depth data from ETOPO dataset[29].

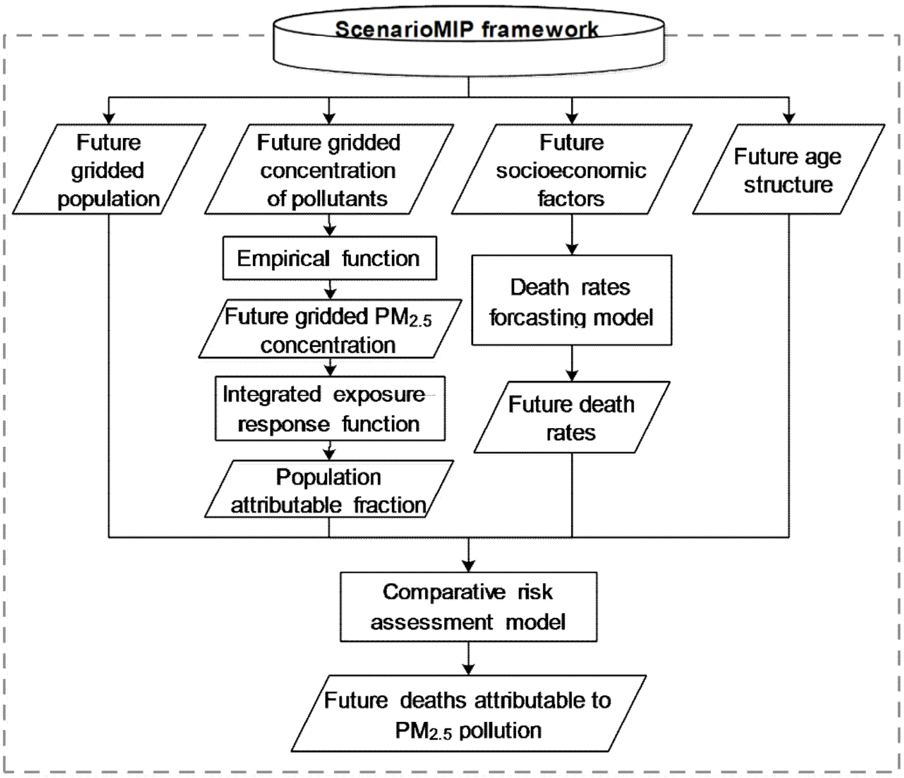

Fig. S2. Flow chart for estimating the future deaths attributable to $PM_{2.5}$ pollution. Deaths attributable to $PM_{2.5}$ pollution were projected by combining an epidemiological model with all driving factors under the ScenarioMIP framework. Population and age structure were derived from an existing dataset, while future $PM_{2.5}$ concentration and the death rate of diseases were estimated in this study. See details in Supplementary Note 3 and Supplementary Note 4.

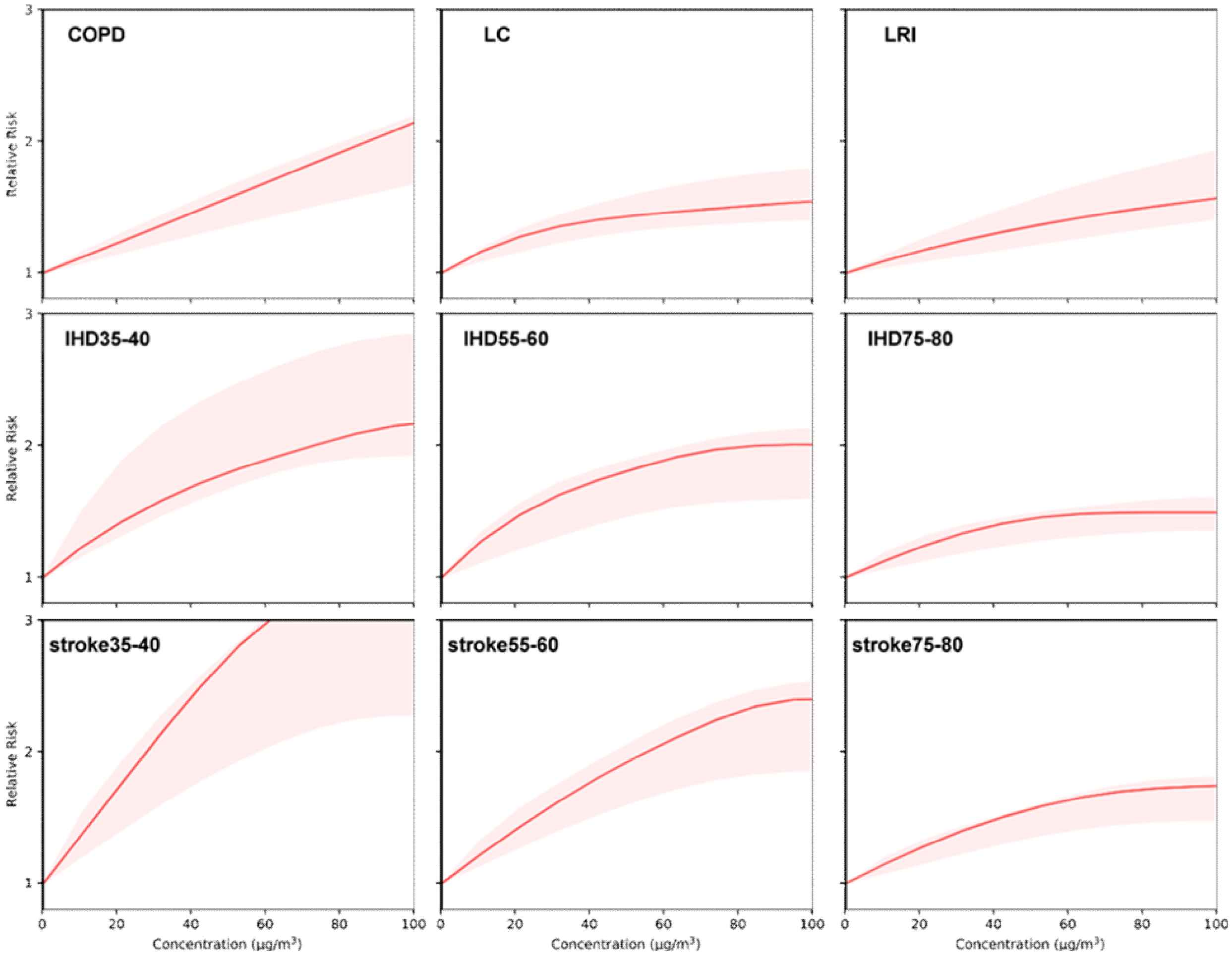

Fig. S3. Exposure-response functions of Bayesian, regularized, trimmed (MR-BRT) model derived from GBD 2019 Risk Factors Collaborators (2020). IHD, COPD, LC and LRI refer to ischemic heart disease, chronic obstructive pulmonary disease, lung cancer, and lower respiratory infection, respectively. 35-40, 55-60 and 75-80 refers to the specific age groups. Shades refers to 95% confidence interval derived from cohort study.

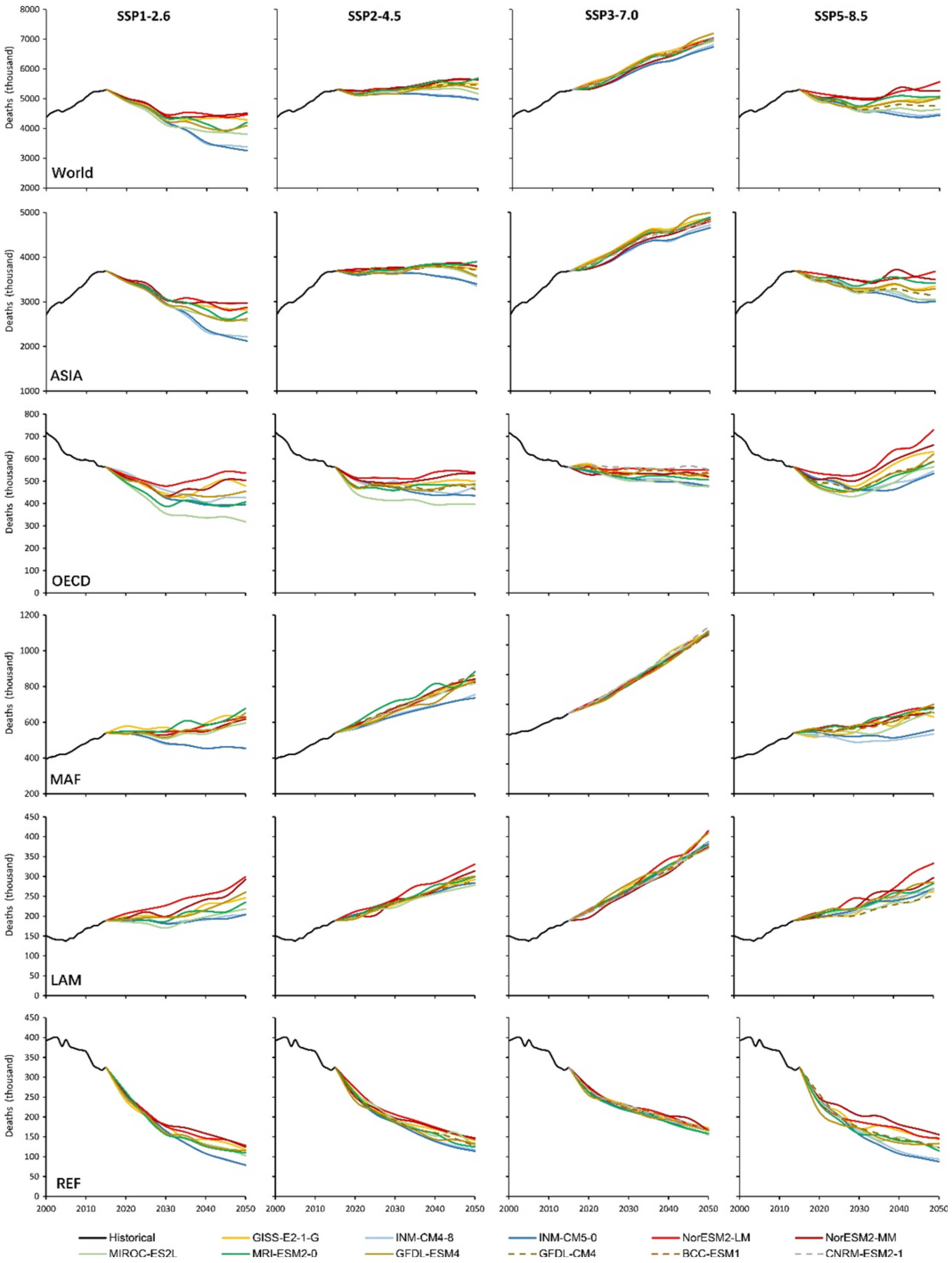

Fig. S4. Trends of deaths attributable to $PM_{2.5}$ pollution by model. The results were estimated based on medium value of exposure-response function and death rate of disease. Please refers to Fig. S1 for detailed definitions of ASIA, OECD, MAF, REF and LAM. Please refers to Table S3 for detailed information of models.

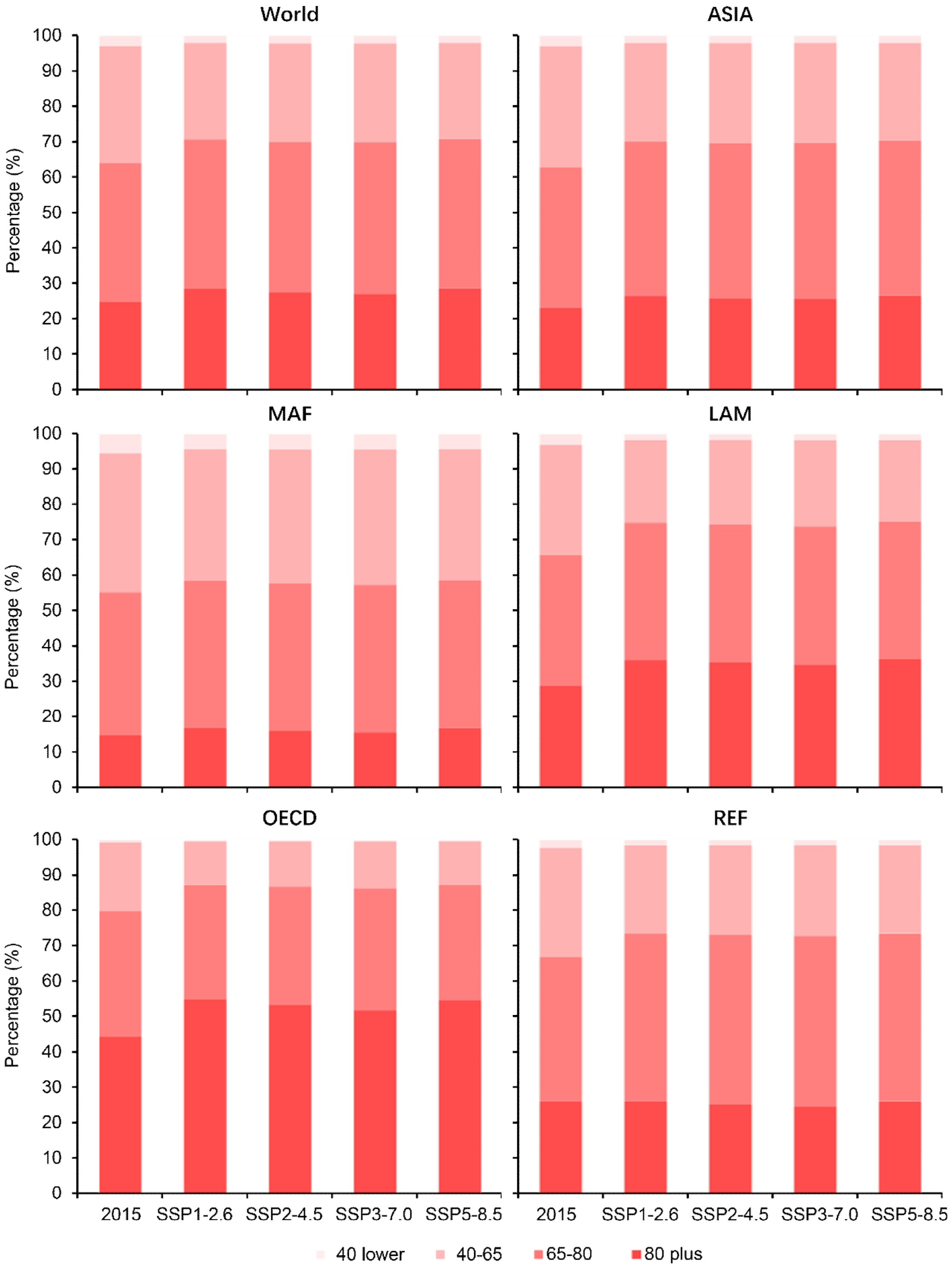

Fig. S5. Trends of deaths attributable to $PM_{2.5}$ pollution by age group. The results were estimated based on multi-model averaged $PM_{2.5}$ concentration, medium value of exposure-response function and death rate of disease. Please refers to Fig. S1 for detailed definitions of ASIA, OECD, MAF, REF and LAM.

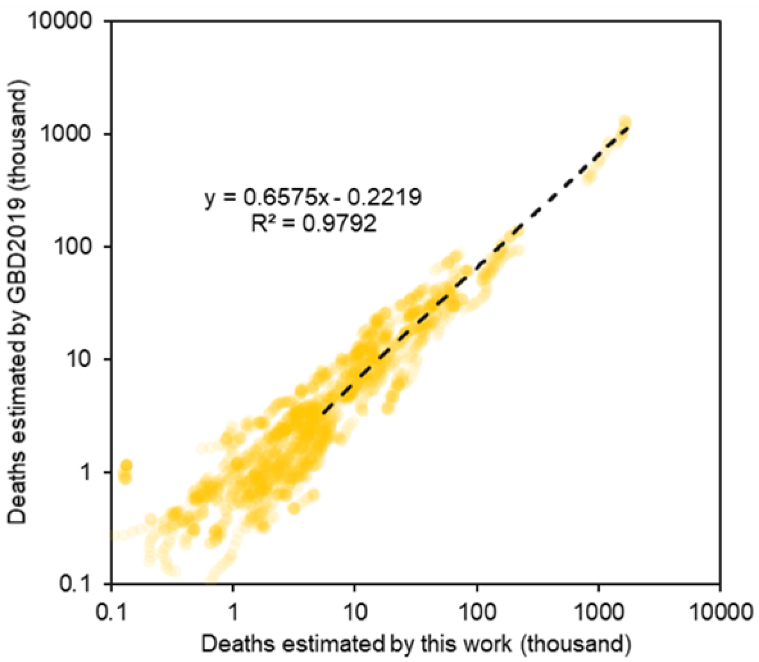

Fig. S6. Comparison of deaths attributable to $PM_{2.5}$ pollution between our estimates and GBD 2019 Risk Factors Collaborators (2020). GBD 2019 results[11] are widely recognized estimates of long-term trend in deaths attributable to $PM_{2.5}$ pollution.

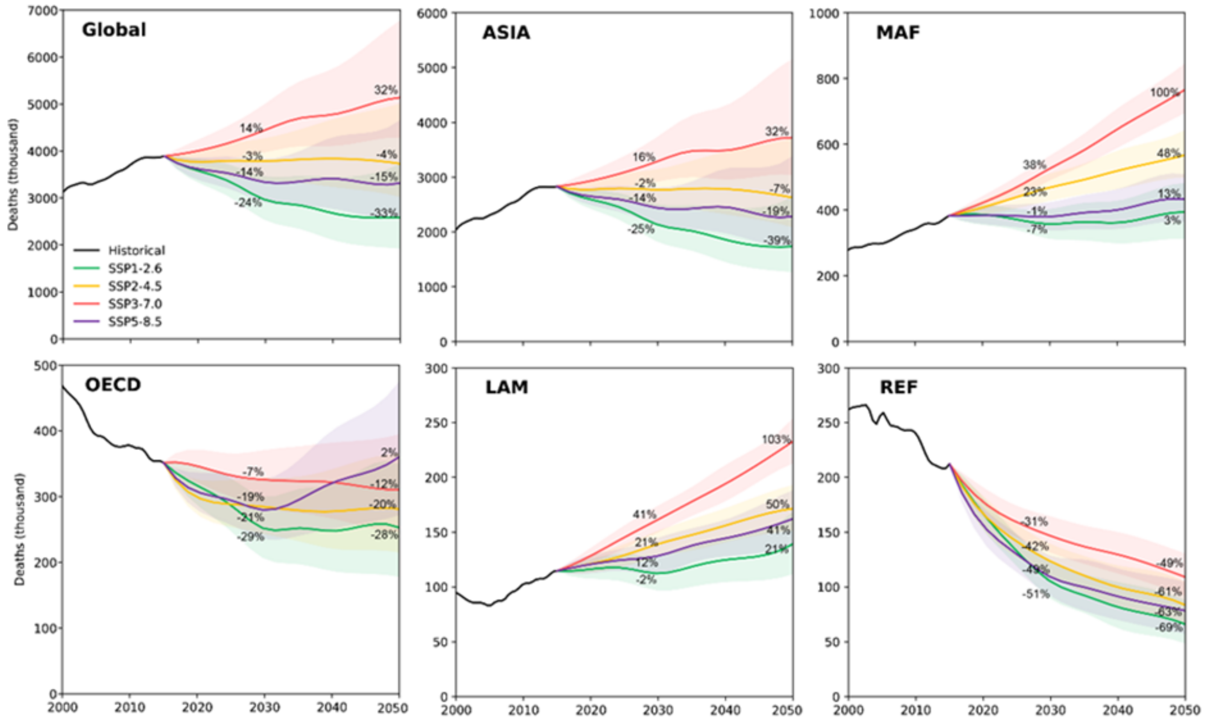

Fig. S7. Changes in DAPP estimated based on the lower value of the exposure-response function. Solid lines represent the average estimates and shading indicates the 95% confidence interval from the uncertainty in future $PM_{2.5}$ concentration (derived from 11 climate and earth system models) and the death rate of diseases (derived from statistic model). Please refers to Fig. S1 for detailed definitions of ASIA, OECD, MAF, REF and LAM.

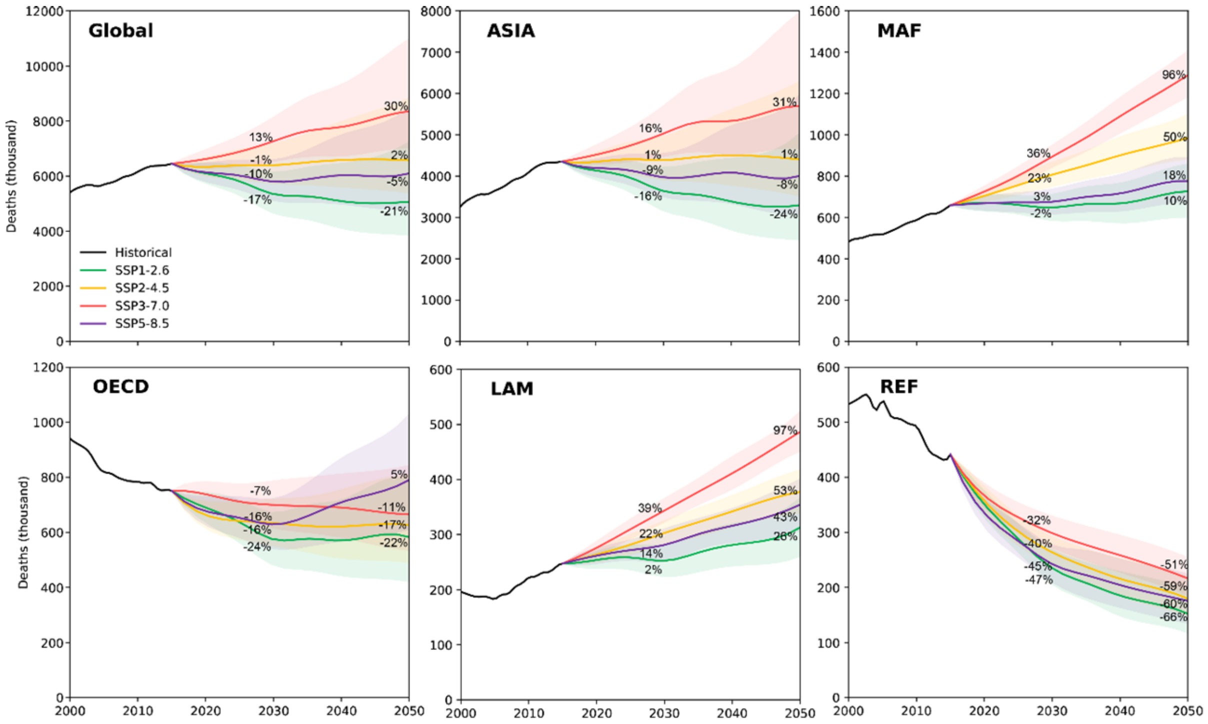

Fig. S8. Changes in DAPP estimated based on the upper value of the exposure-response function. Solid lines represent the average estimates and shading indicates the 95% confidence interval from the uncertainty in future $PM_{2.5}$ concentration (derived from 11 climate and earth system models) and the death rate of diseases (derived from statistic model). Please refers to Fig. S1 for detailed definitions of ASIA, OECD, MAF, REF and LAM.

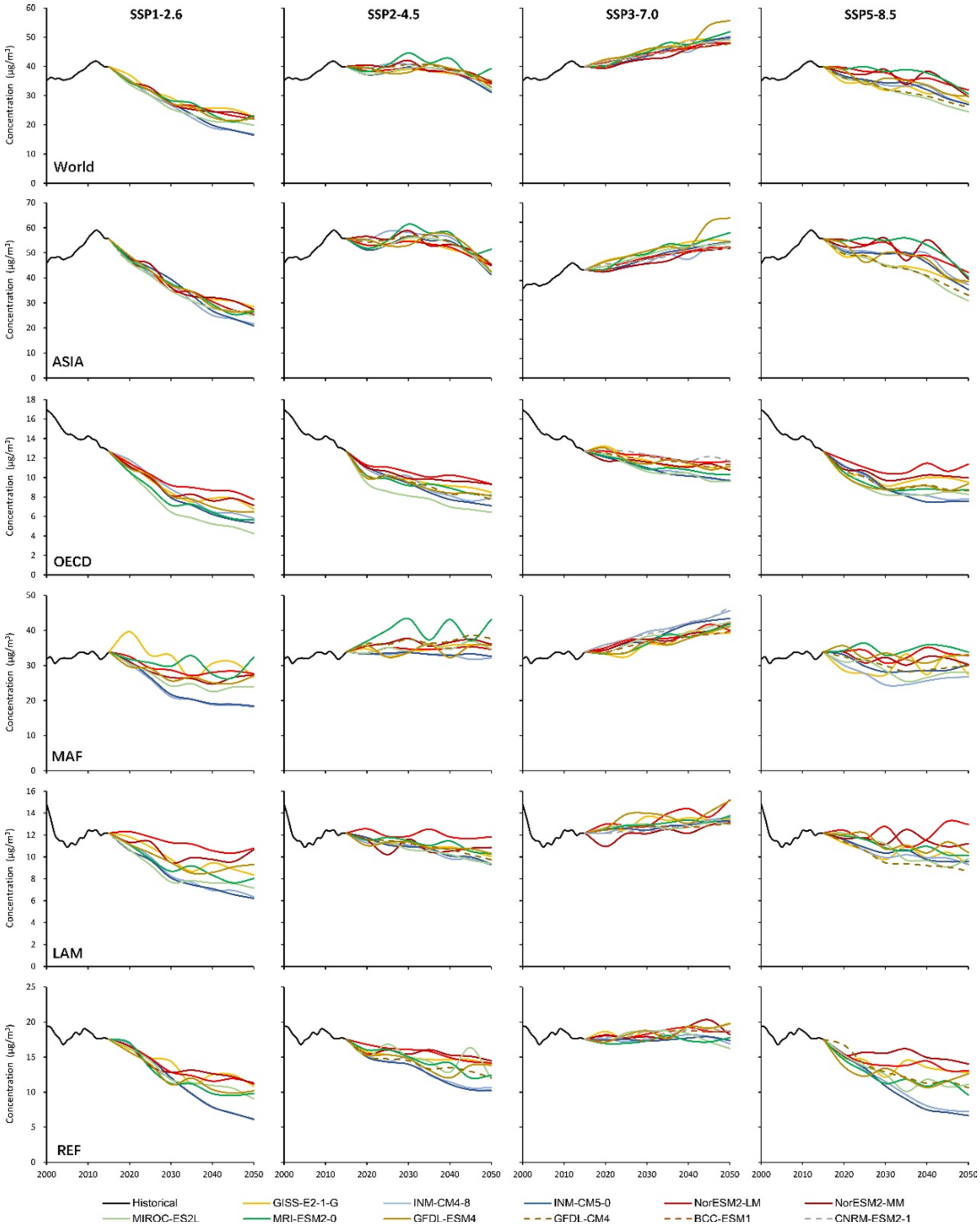

Fig. S9. Trends of population-weighted $PM_{2.5}$ concentration by model. The $PM_{2.5}$ concentration is weighted by population to indicate the overall exposure. Please refers to Fig. S1 for detailed definitions of ASIA, OECD, MAF, REF and LAM. Please refers to Table S3 for detailed information of models.

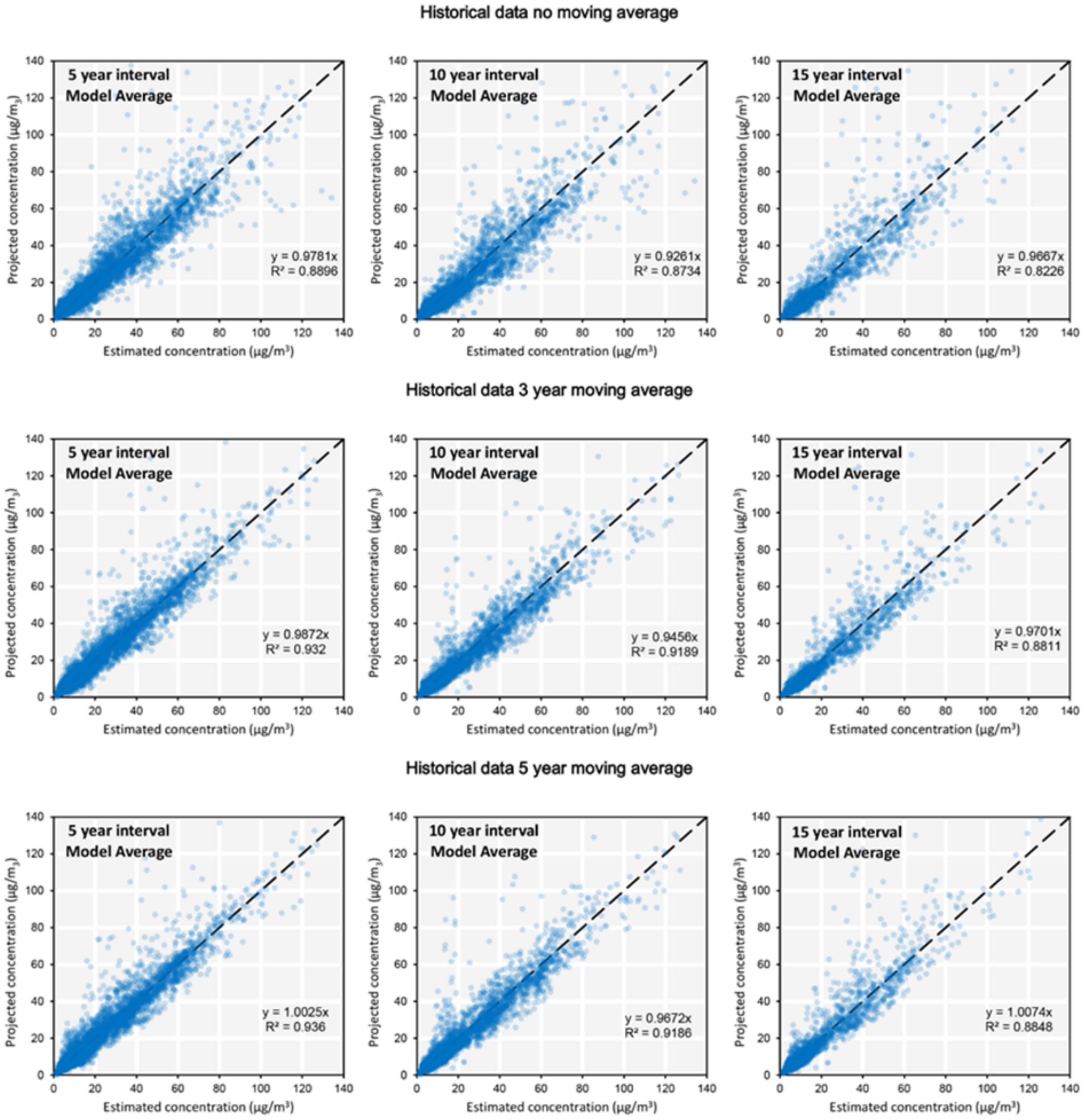

Fig. S10. Validation of projected $PM_{2.5}$ concentration (multi-model average). The projected $PM_{2.5}$ concentration was extrapolated based on historical data with three different moving average strategies (no moving average, 3 year moving average, and 5 year moving average), with three time-spans of 5, 10, and 15 years.

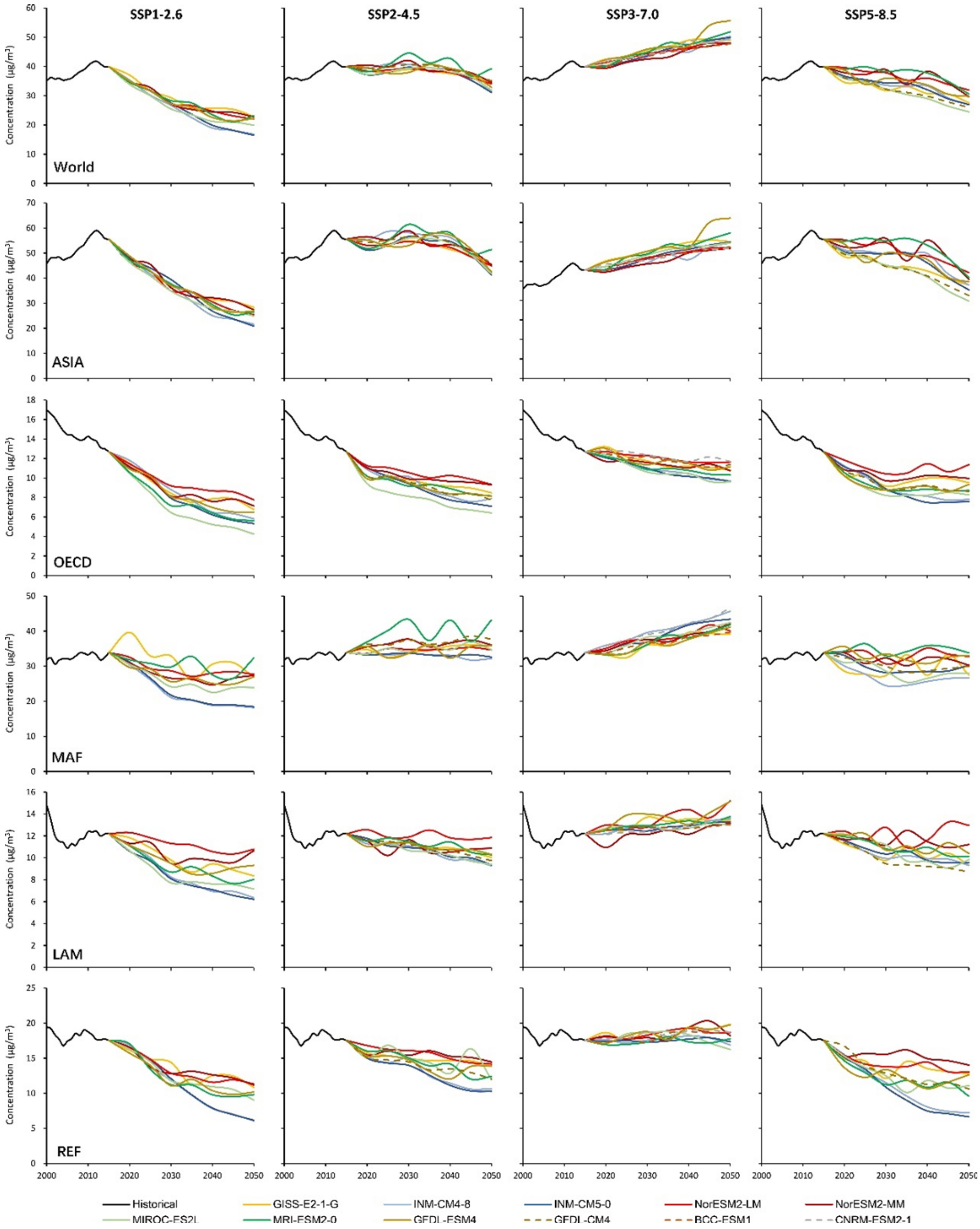

Fig. S11. Model-specific $PM_{2.5}$ concentration. Please refers to Fig. S1 for detailed definitions of ASIA, OECD, MAF, REF and LAM.

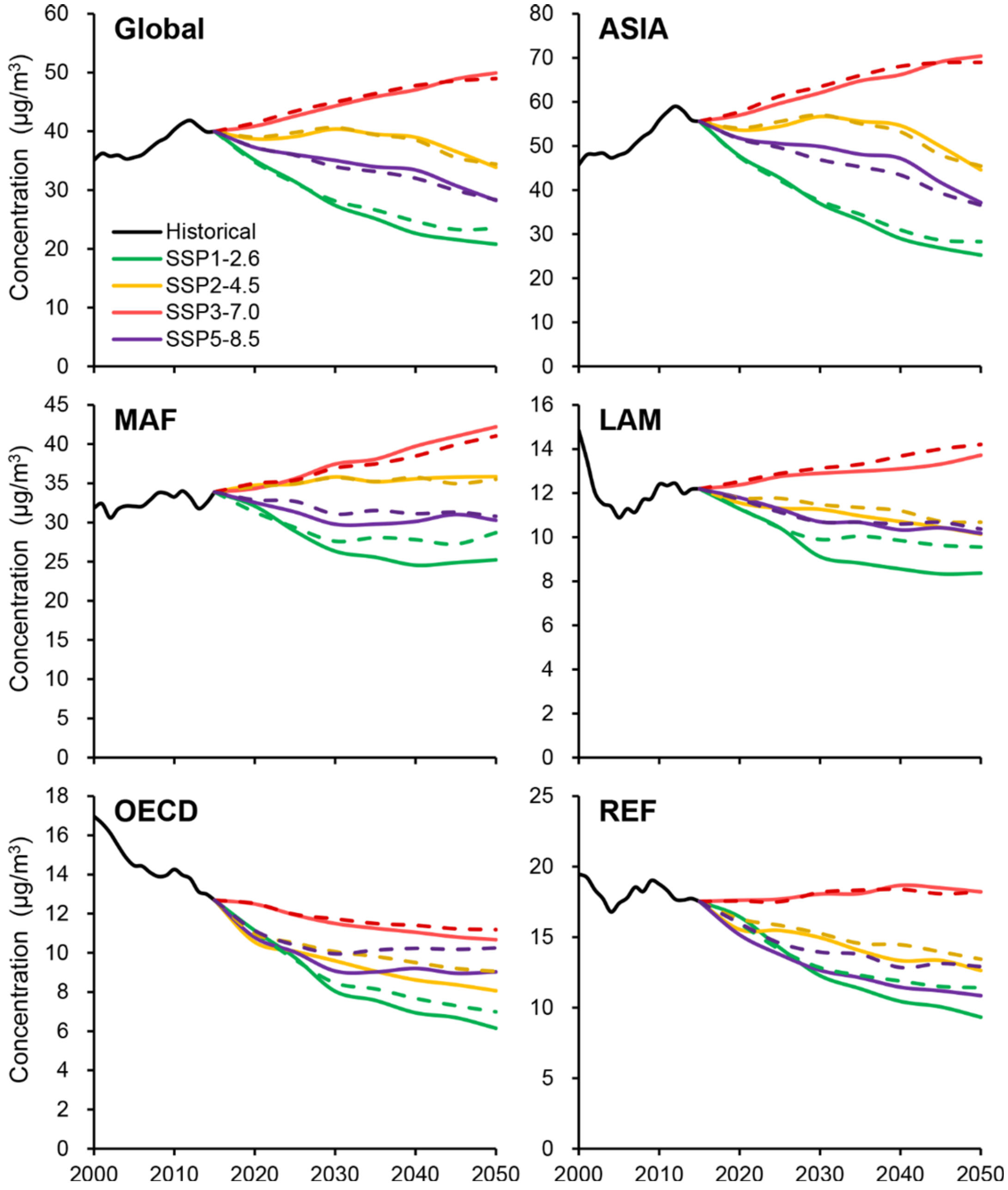

Fig. S12. Comparison of multi-model averaged $PM_{2.5}$ concentration between our projection (solid line) and Turnock et al (2020) (dashed line). Please refers to Fig. S1 for detailed definitions of ASIA, OECD, MAF, REF and LAM.

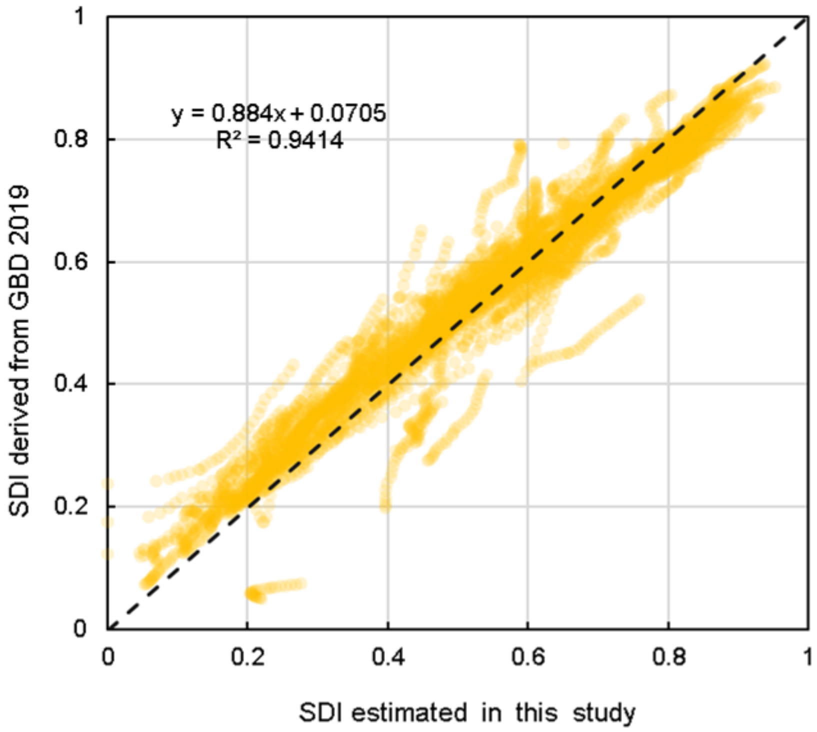

Fig. S13. Comparison of historical socio-demographic index (SDI) between our study and GBD 2019. GBD 2019 results[11] are widely recognized estimates of long-term trends in SDI.

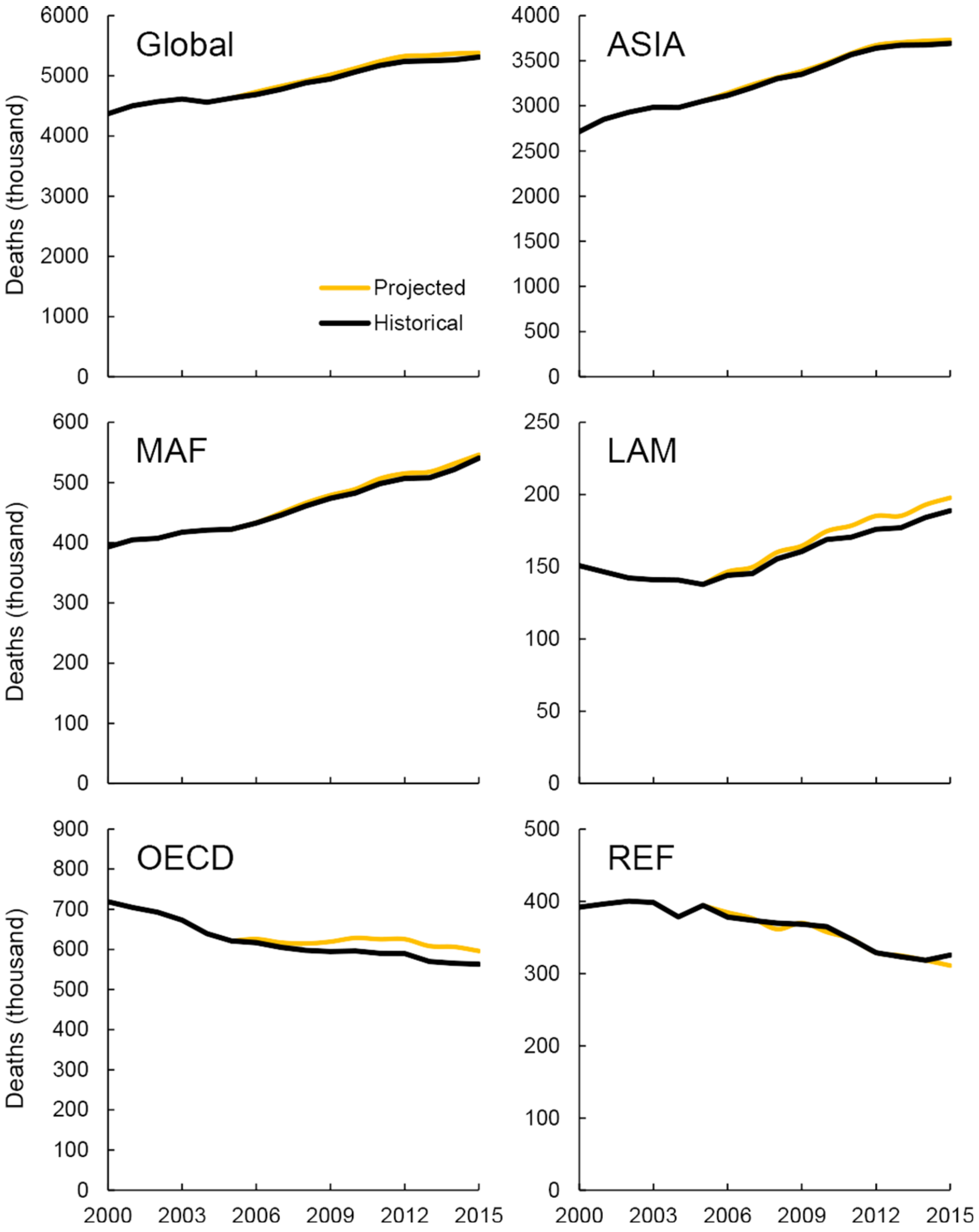

Fig. S14. Validation of the death rate projection. Please refers to Fig. S1 for detailed definitions of ASIA, OECD, MAF, REF and LAM.

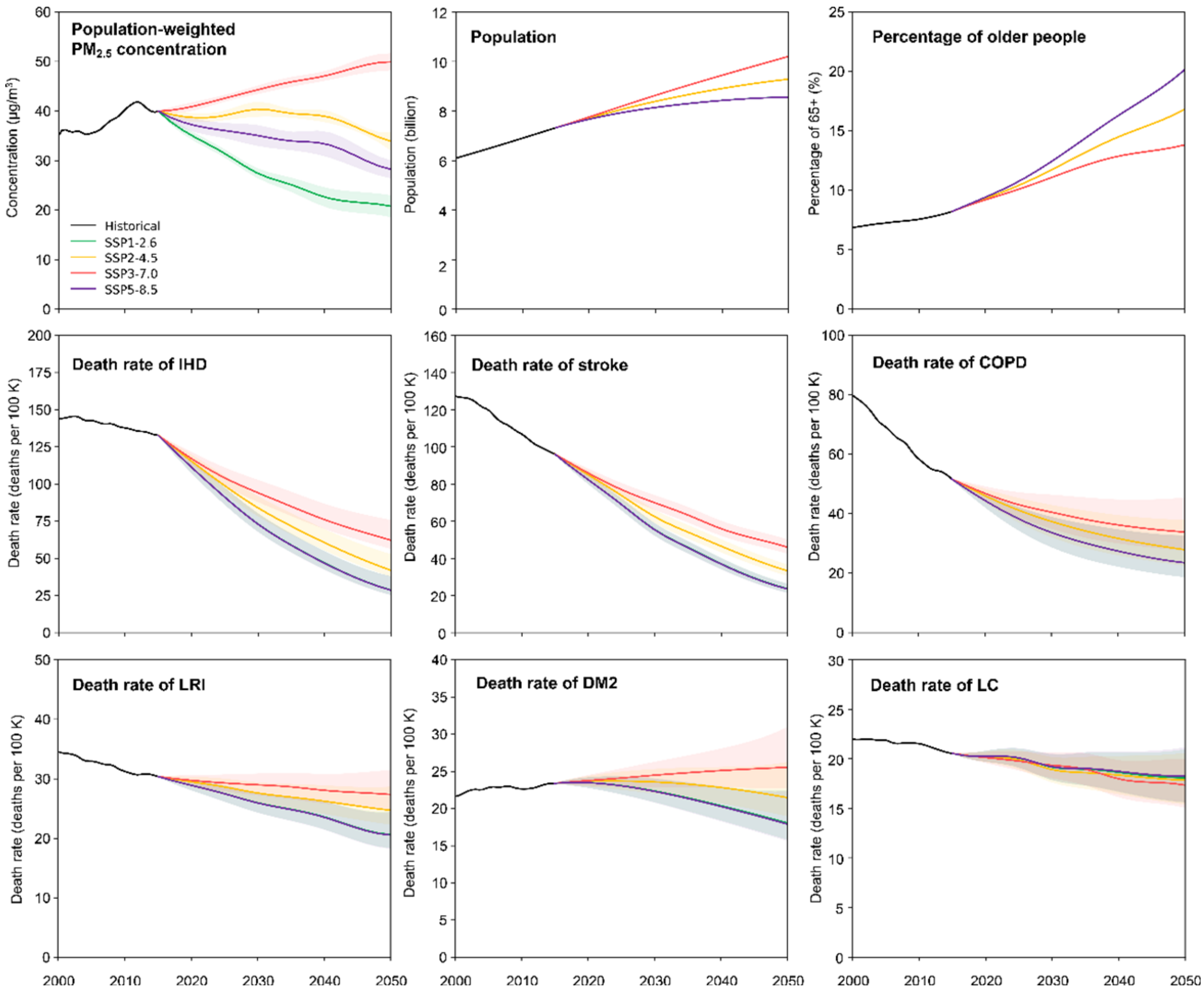

Fig. S15. Global changes in the driving factors of DAPP. The $PM_{2.5}$ concentration is weighted by population to indicate the overall exposure at the global scale. IHD, COPD, LC, LRI, and DM2 refer to ischemic heart disease, chronic obstructive pulmonary disease, lung cancer, lower respiratory infection, and diabetes mellitus type 2, respectively. The death rate of diseases were age-standardized to reflect the healthcare standard without influence of changes in age structure. Shading indicates the 95% confidence interval, see details in Supplementary Note 3-4.

Population weighted $PM_{2.5}$ concentration

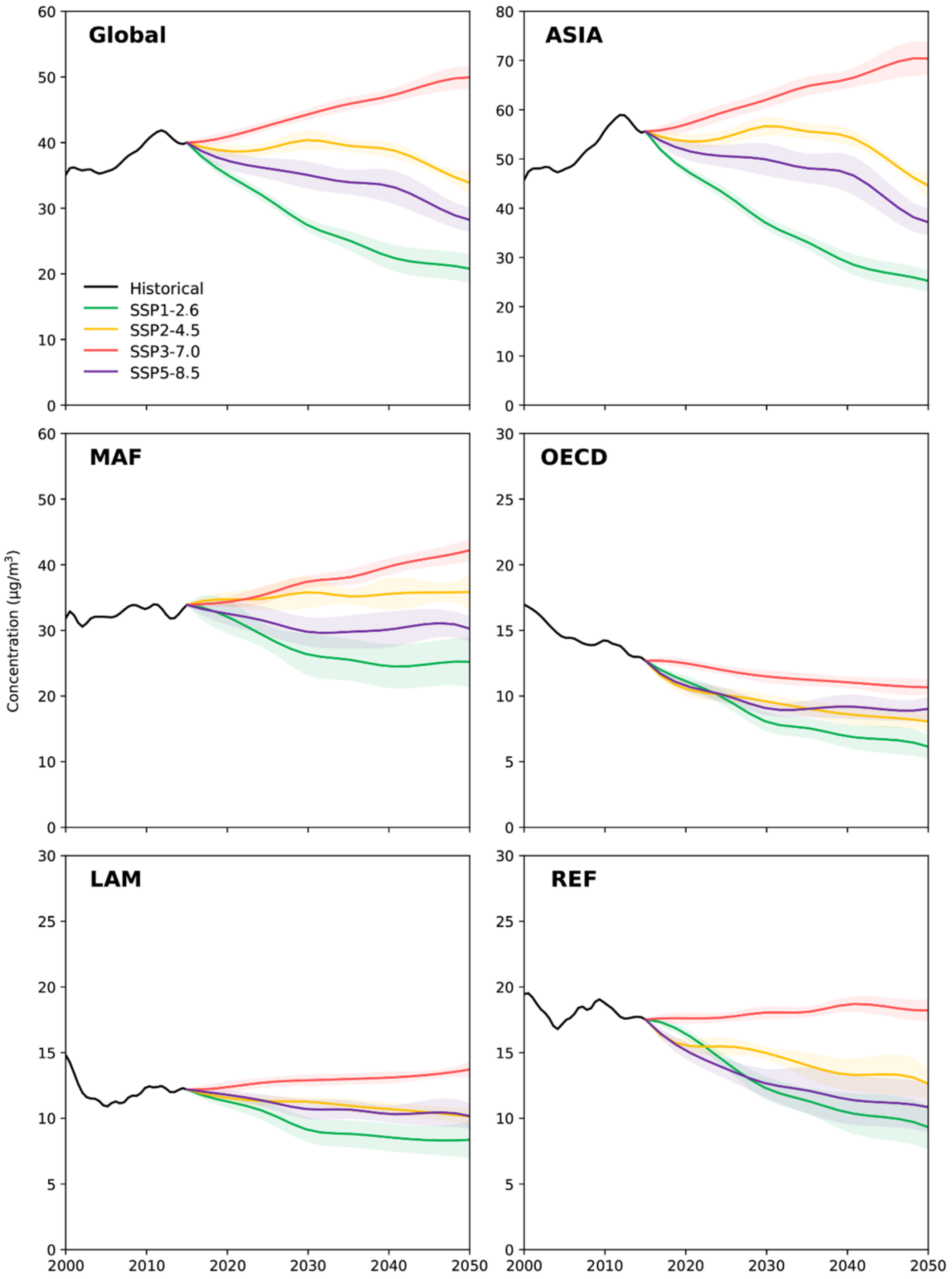

(continued on next page)

Population

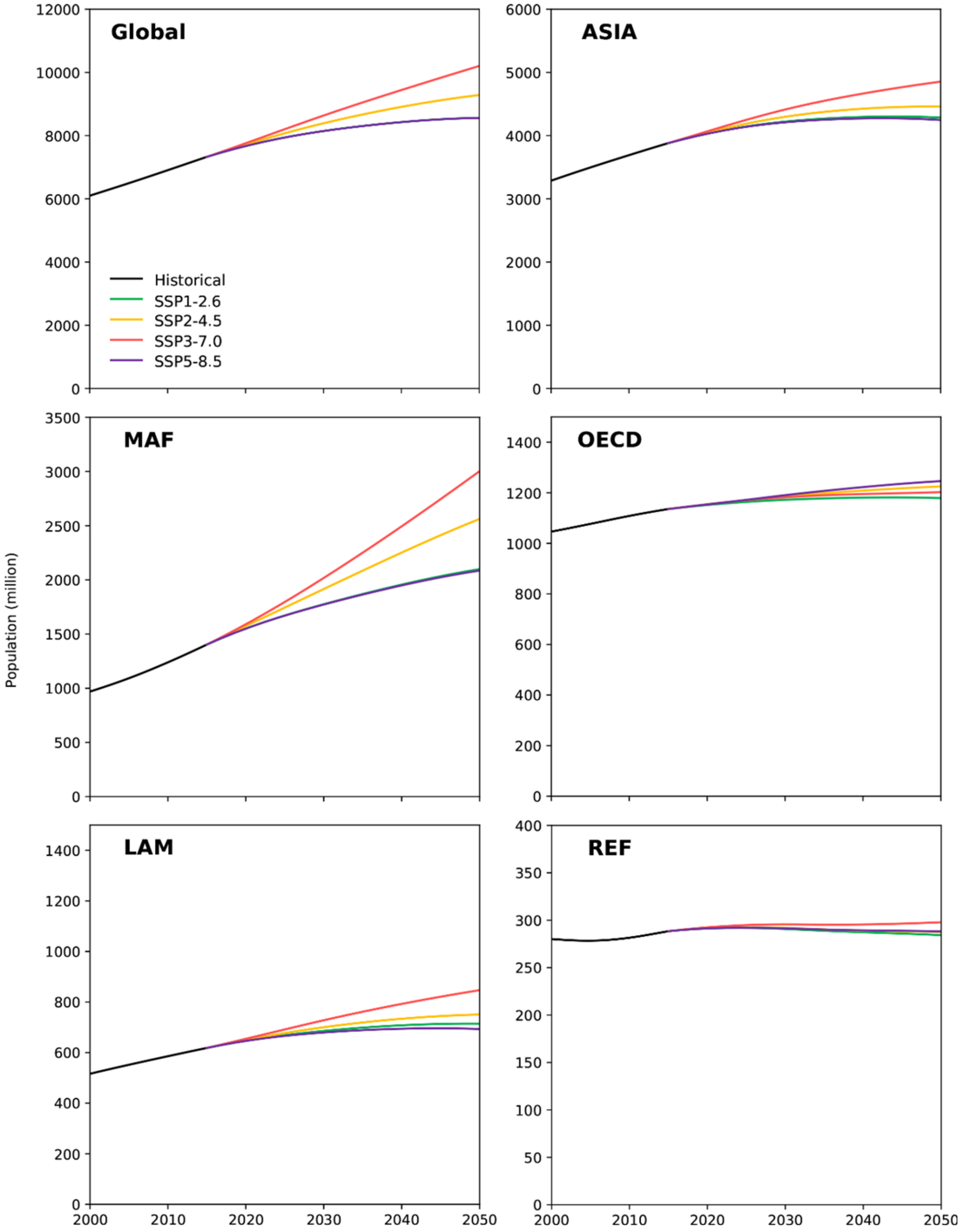

(continued on next page)

Percentage of older people (65+)

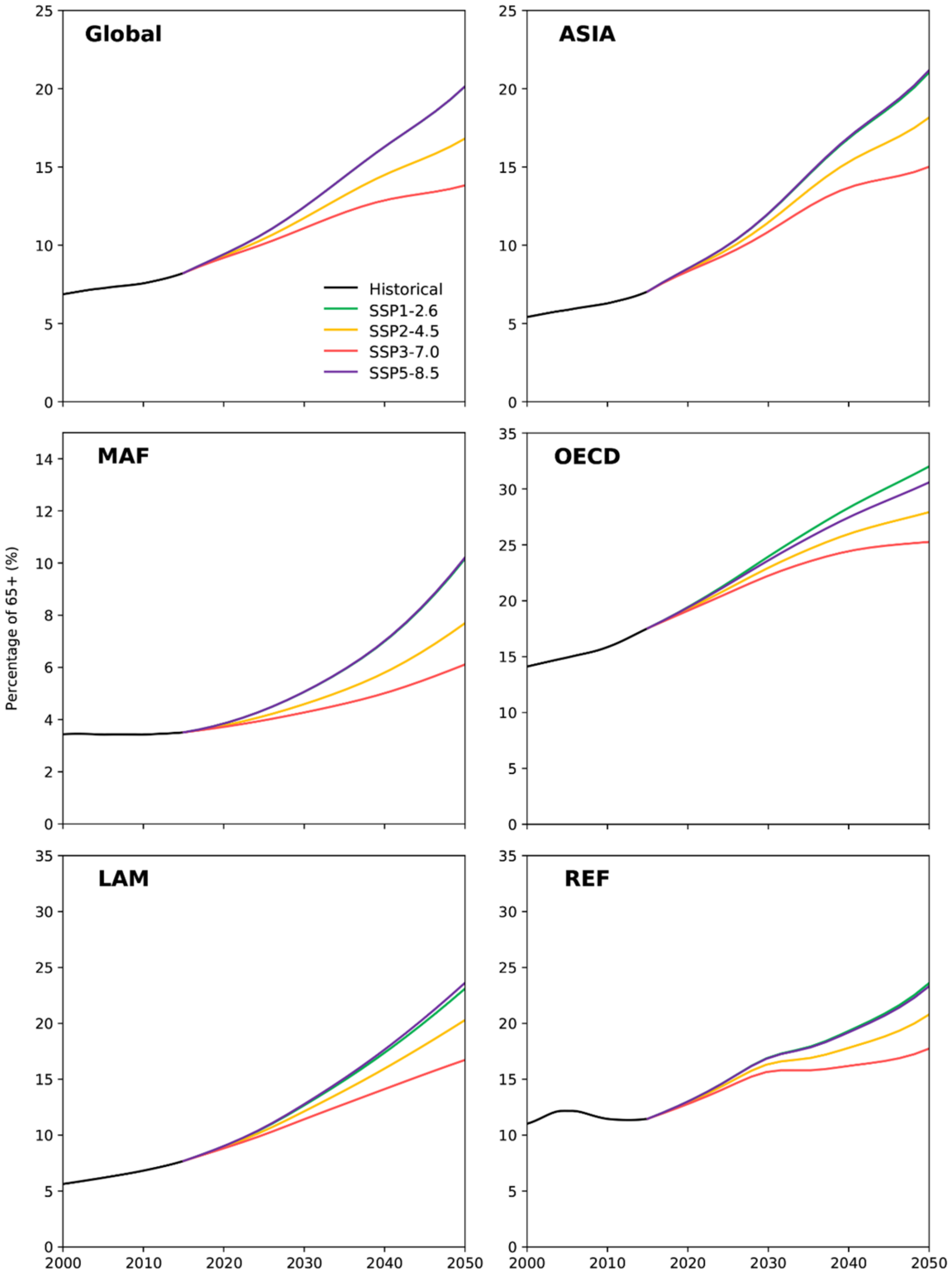

(continued on next page)

Age-standardized death rate of IHD

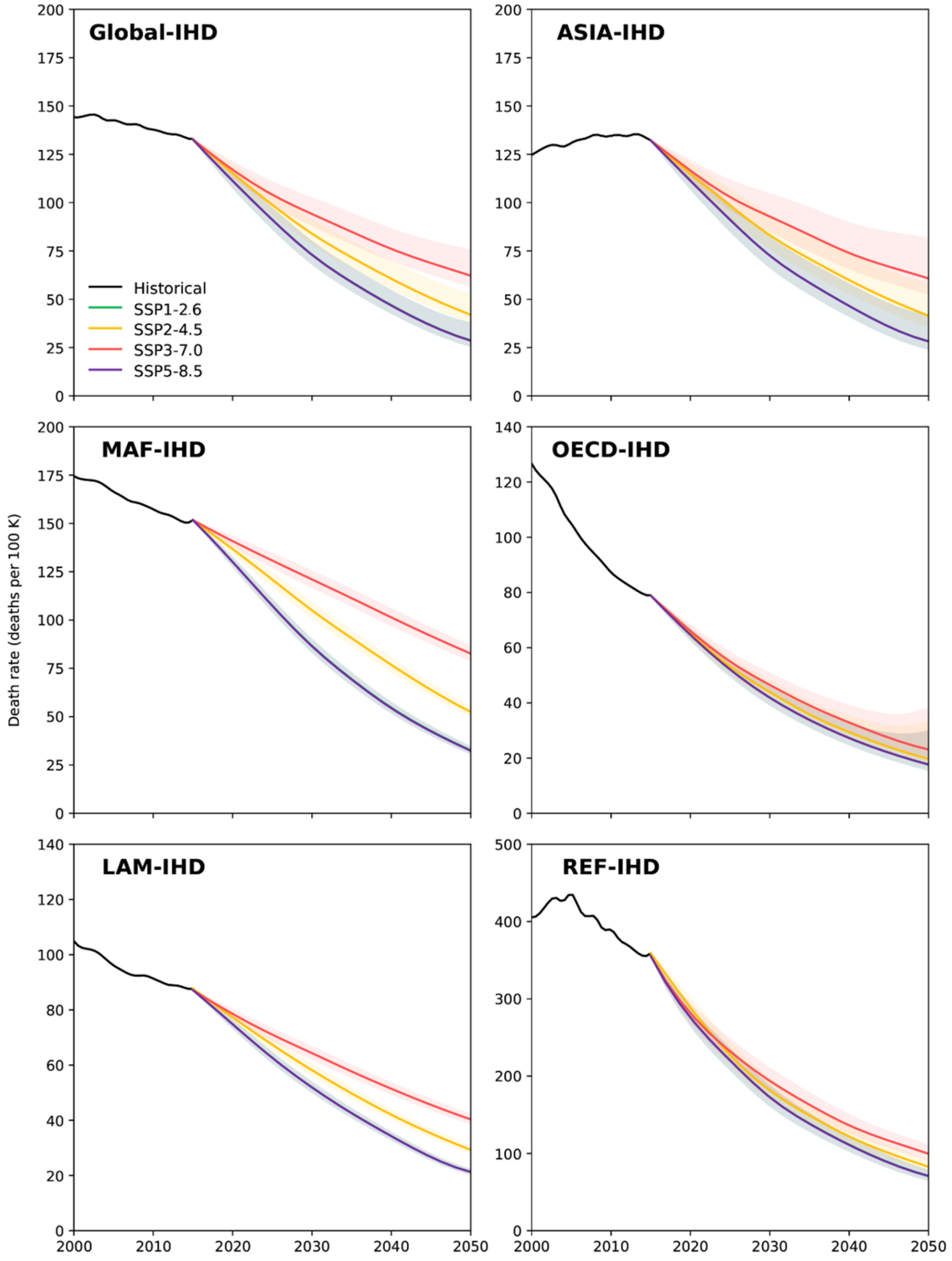

(continued on next page)

Age-standardized death rate of stroke

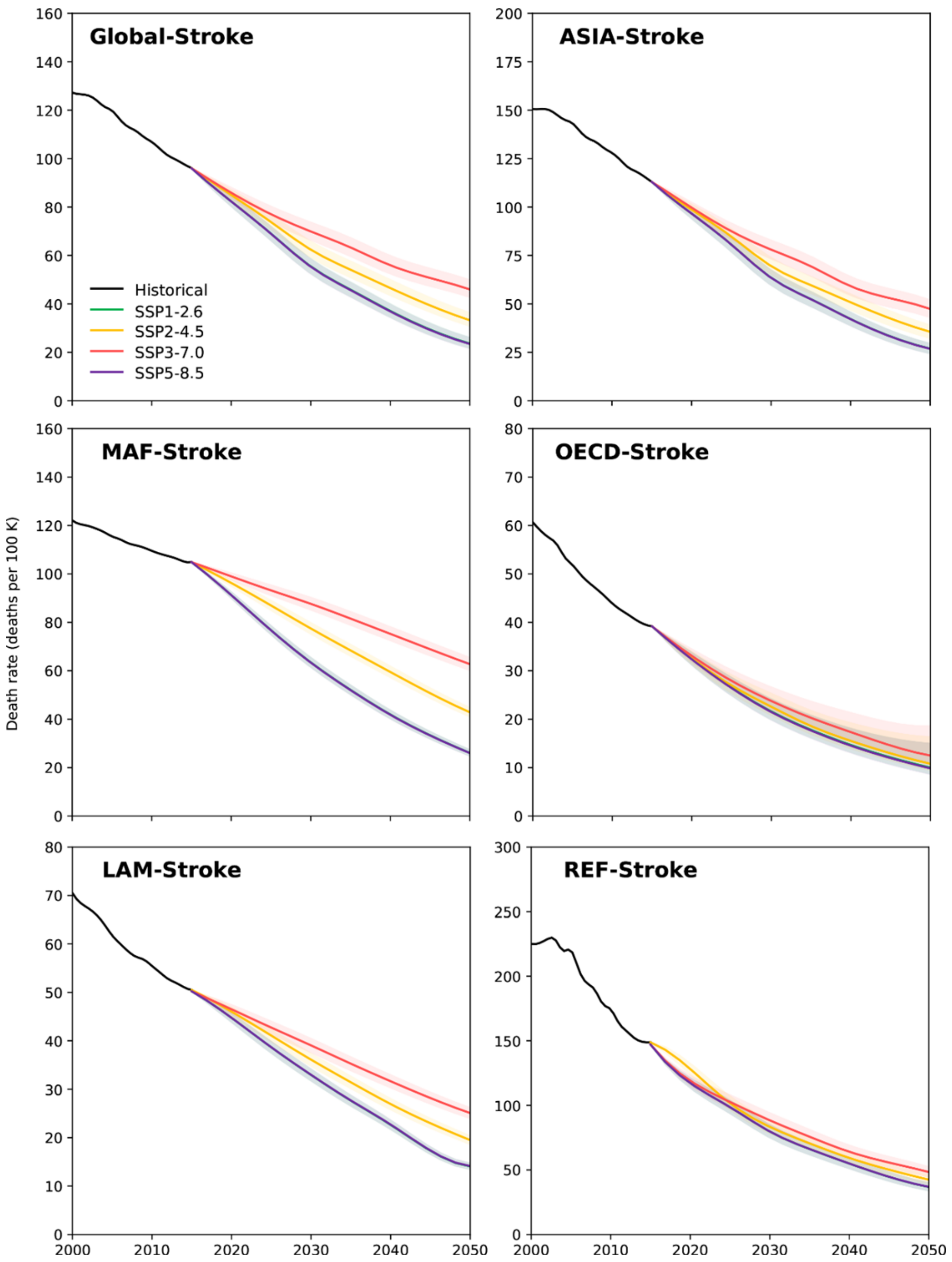

(continued on next page)

Age-standardized death rate of LRI

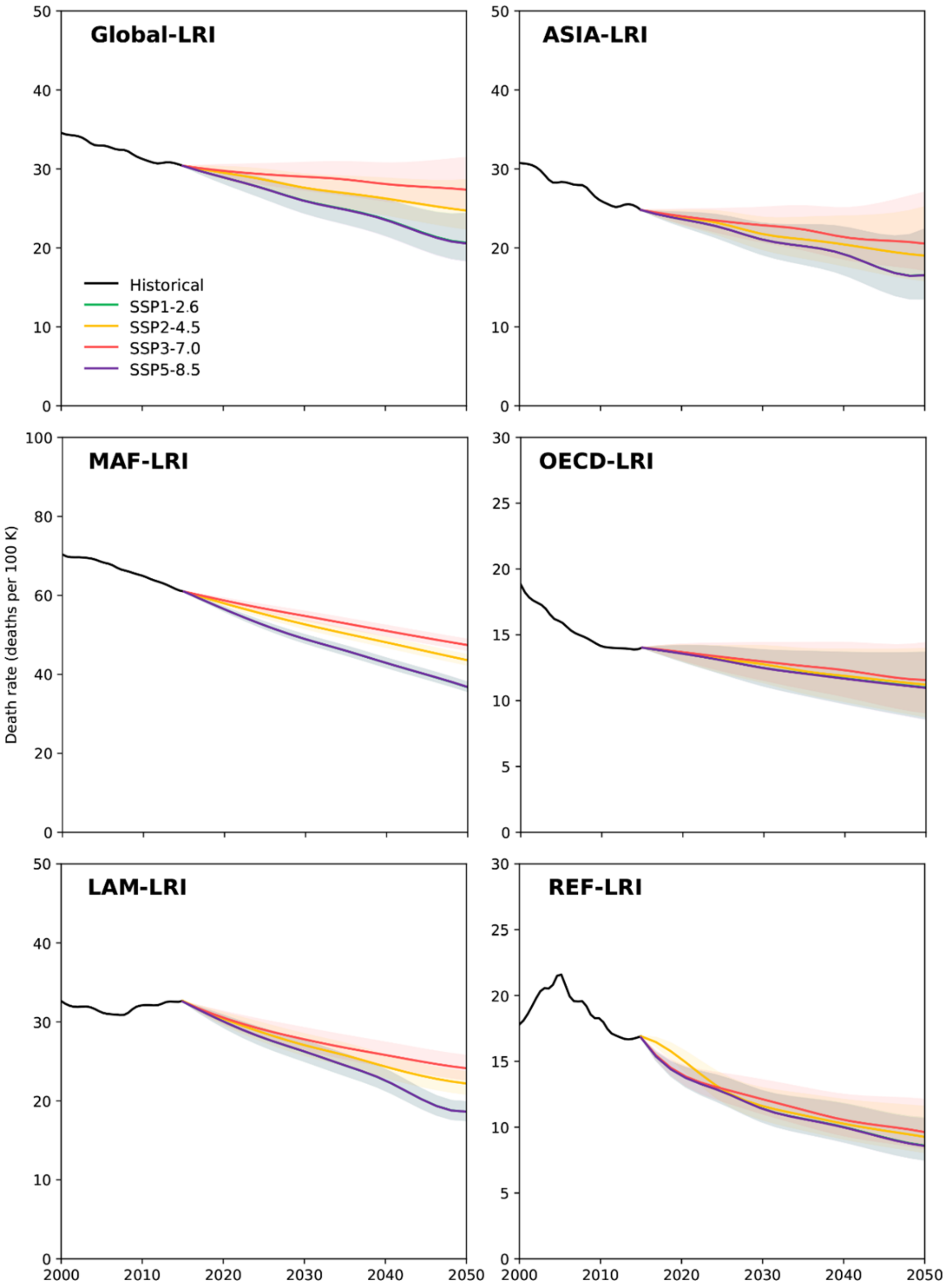

(continued on next page)

Age-standardized death rate of DM2

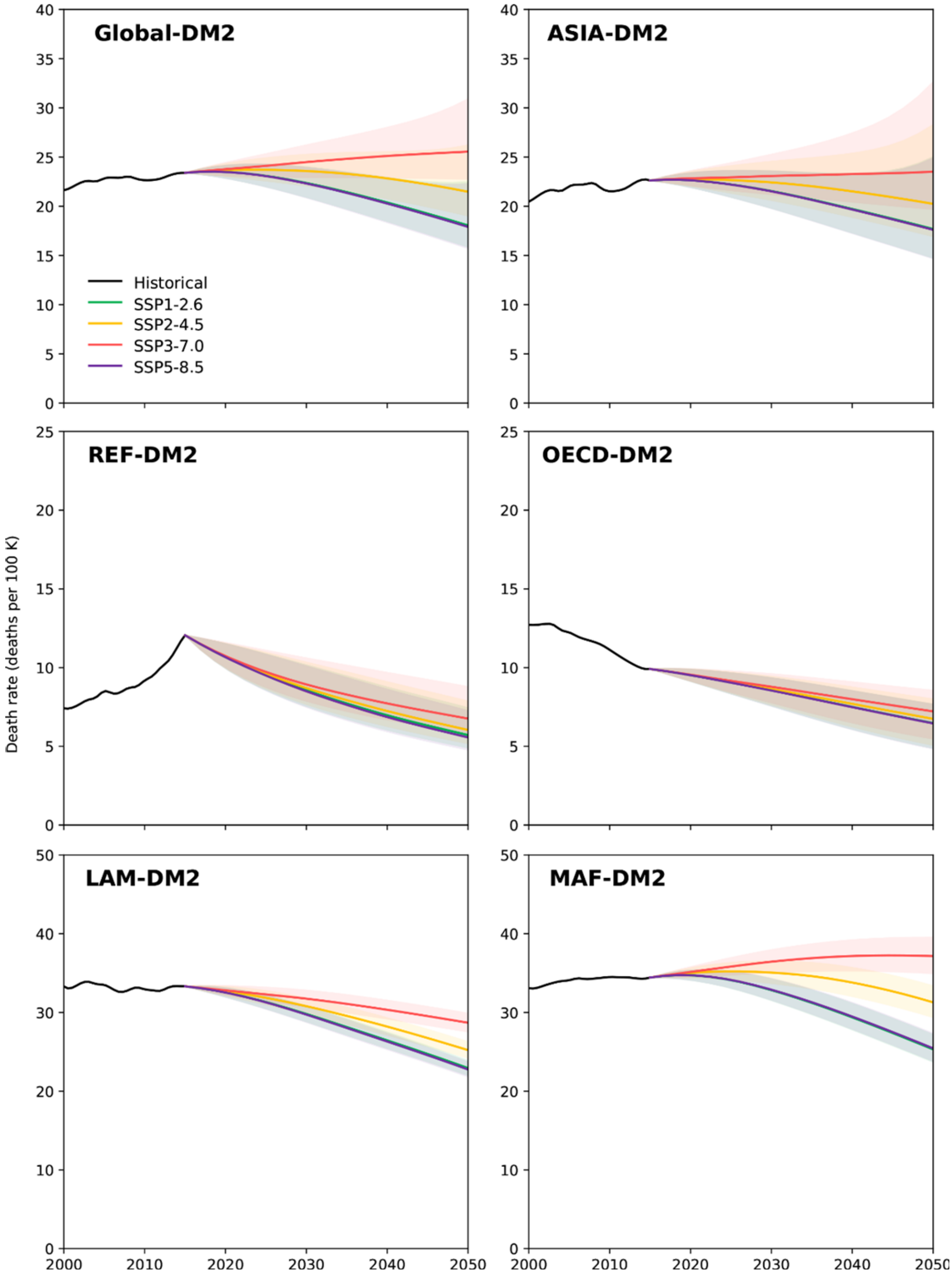

(continued on next page)

Age-standardized death rate of LC

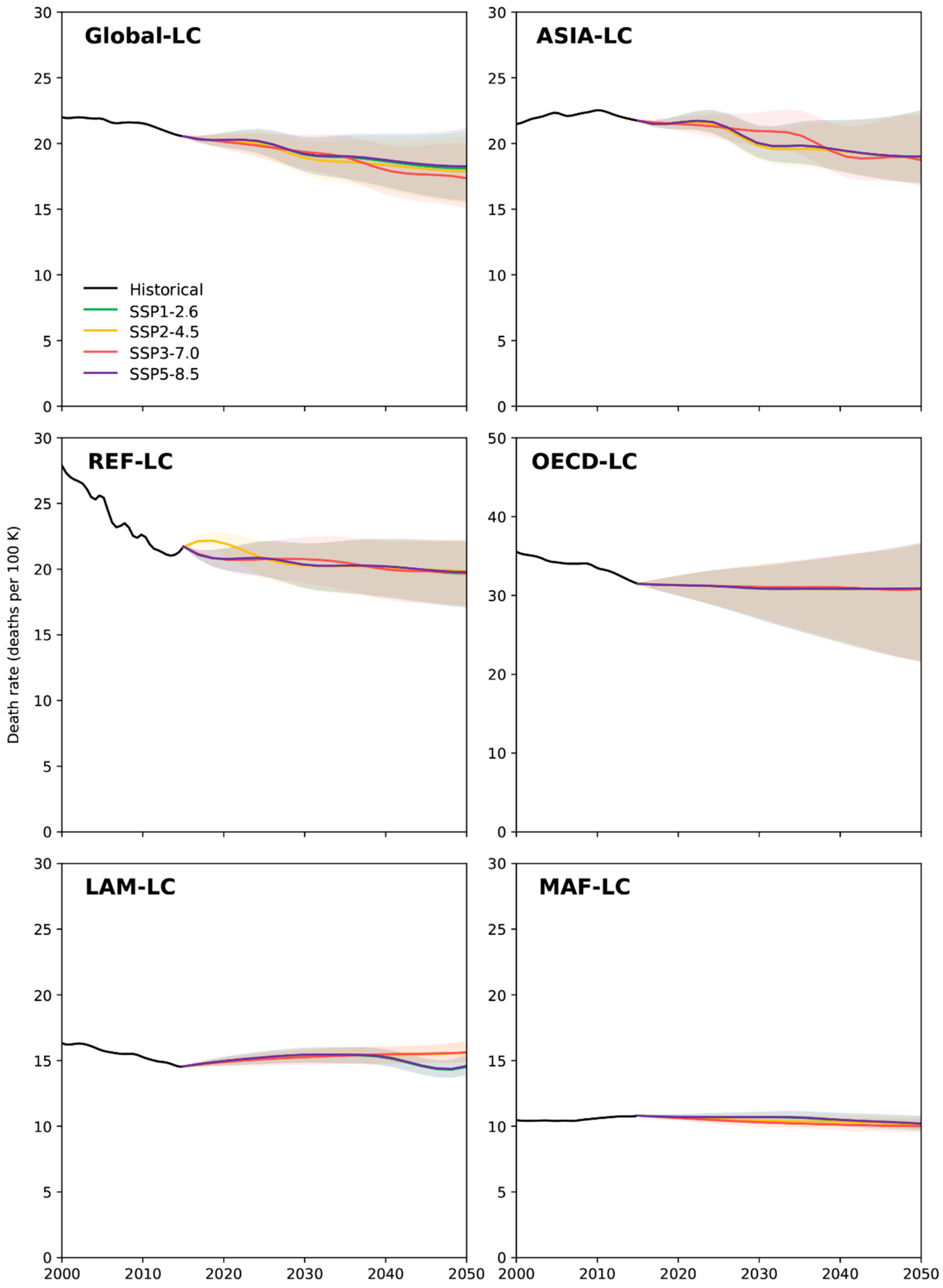

Fig. S16. Region-specific changes in the driving factors of DAPP. IHD, COPD, LC and LRI refer to ischemic heart disease, chronic obstructive pulmonary disease, lung cancer, and lower respiratory infection, respectively. 35-40, 55-60 and 75-80 refers to the specific age groups. Shades refers to 95% confidence interval. Please refers to Fig. S1 for detailed definitions of ASIA, OECD, MAF, REF and LAM.

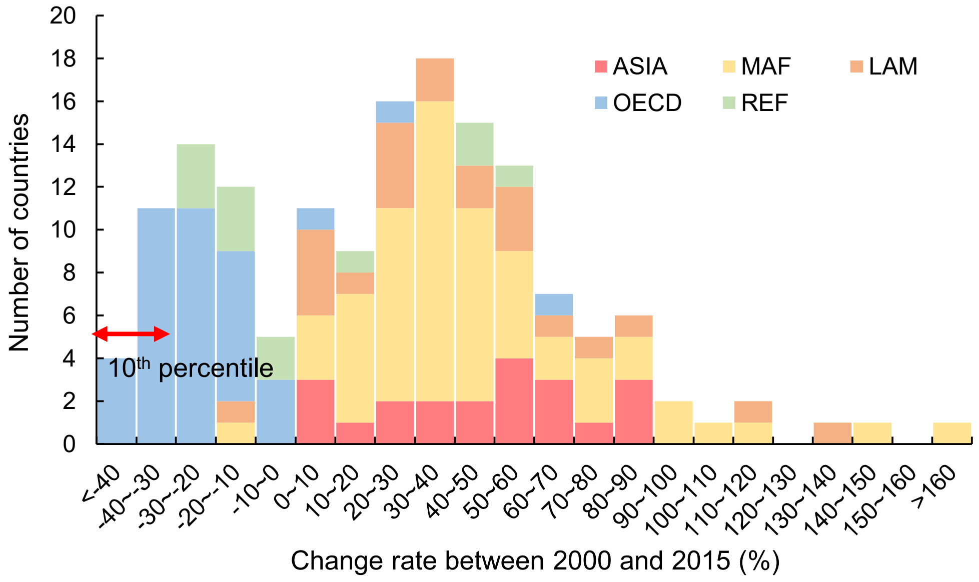

Fig. S17. Histogram for historical change rate in DAPP between 2000 and 2015. The 10th percentile of the reduction in DAPP for countries was around 30%. Please refers to Fig. S1 for detailed definitions of ASIA, OECD, MAF, REF and LAM.

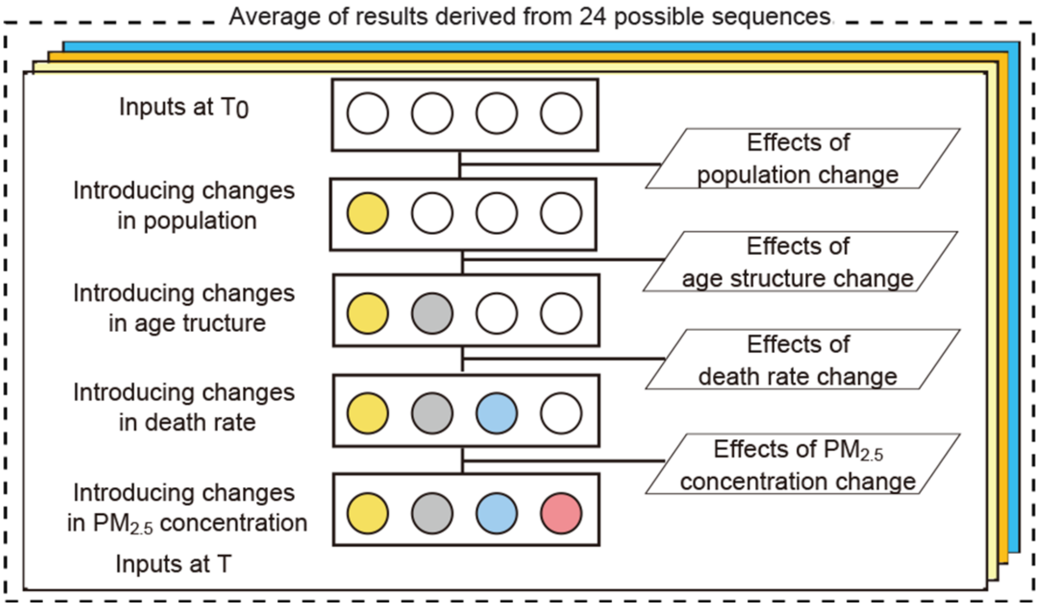

Fig. S18. Framework of the decomposition analysis. The matrix of colored dots indicates specific combinations of driving factors. Yellow, gray, blue and red dots refer to the changes in population, age structure, death rate and $PM_{2.5}$ concentration, respectively. T_0 and T represent the base year and target year, respectively.

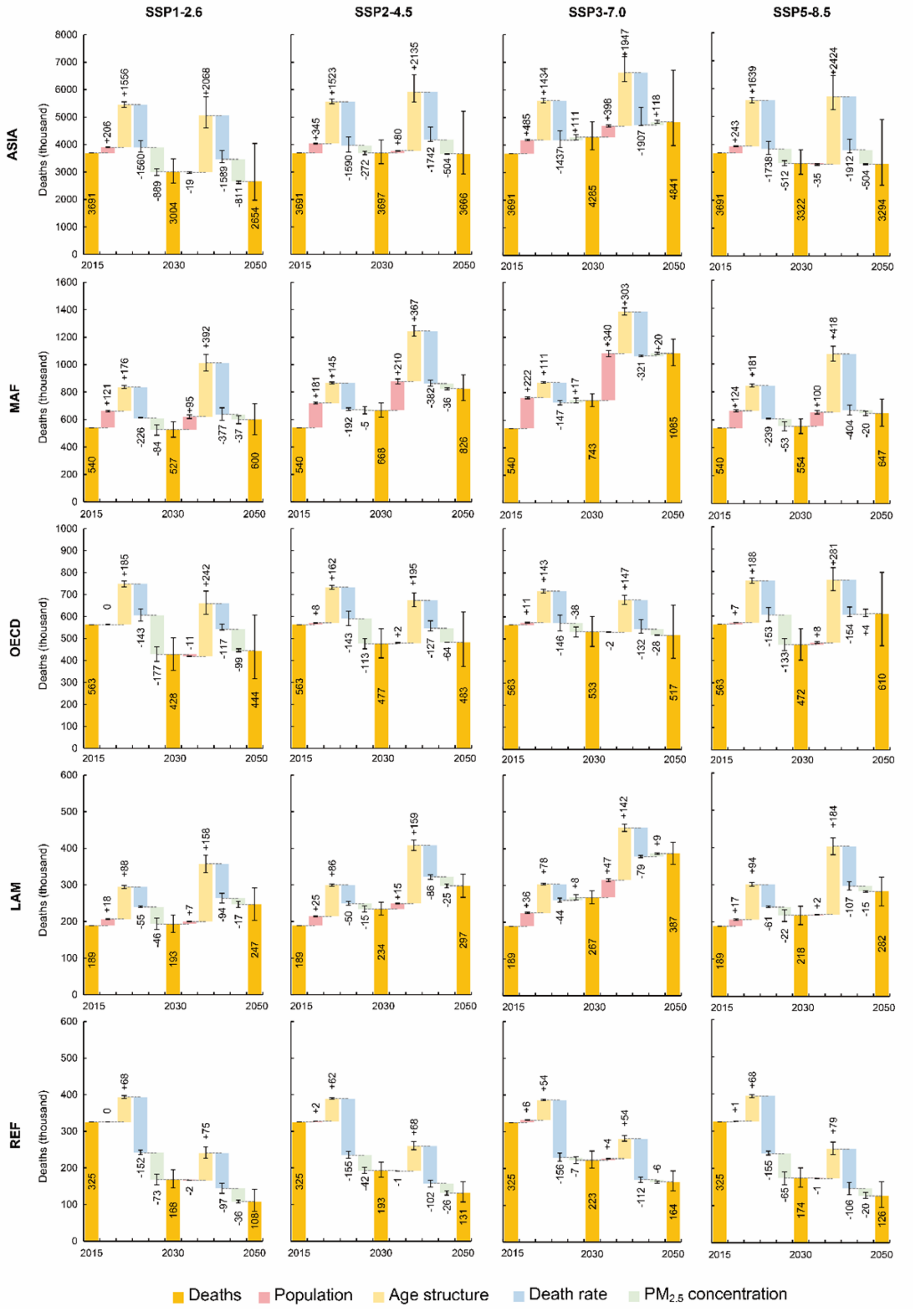

Fig. S19. Region-specific effect of different driving factors on the changes in DAPP. These plots show the cumulative effect of four factors: population, age structure, death rate and $PM_{2.5}$ concentration. The data represented the mean value. Error bars represent 95% confidence intervals from the uncertainty in future $PM_{2.5}$ concentration future $PM_{2.5}$ concentration (derived from 11 climate and earth system models) and the death rate of diseases (derived from statistic model), see details in Supplementary Note 3-4. Please refers to Fig. S1 for detailed definitions of ASIA, OECD, MAF, REF and LAM.

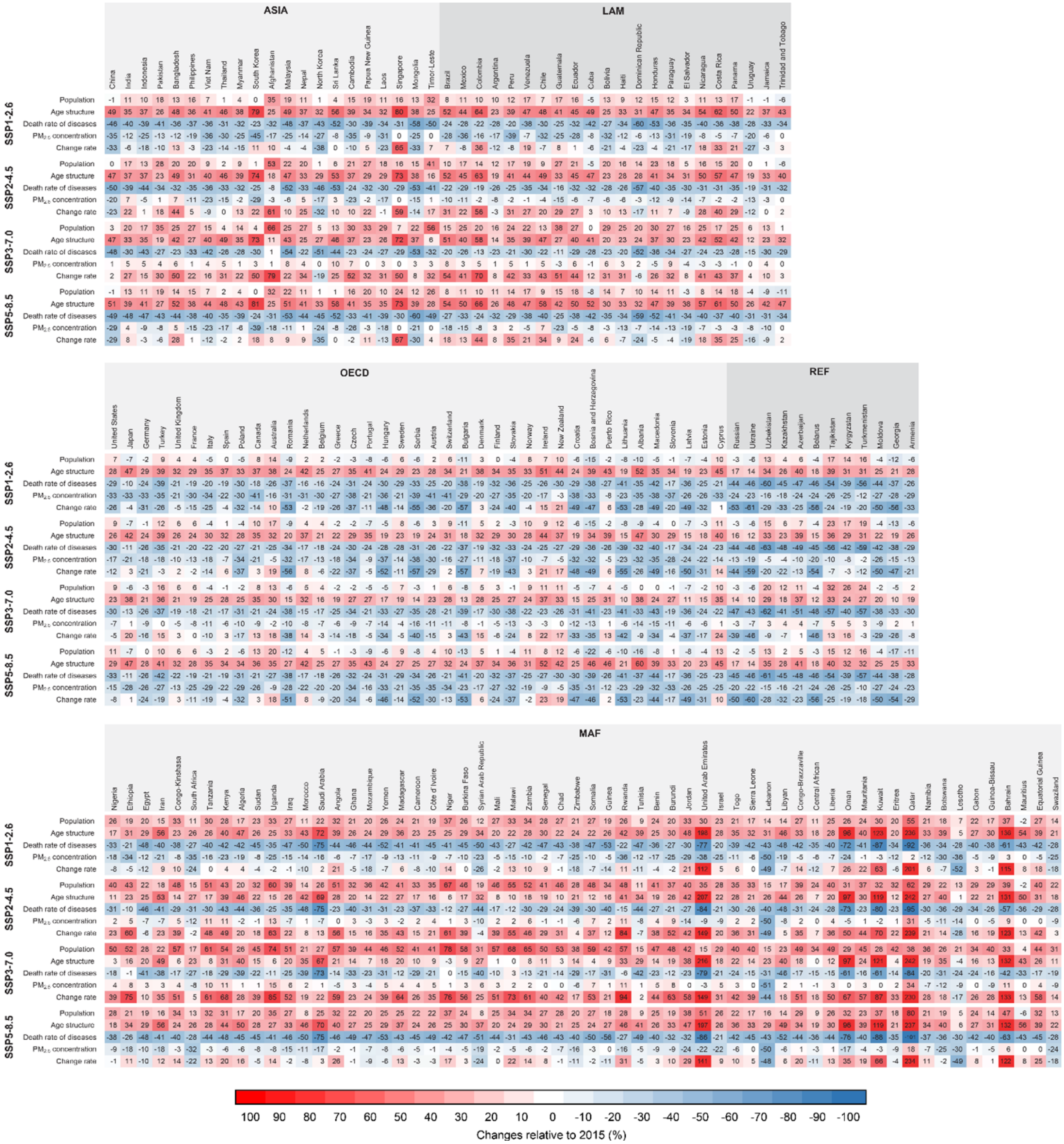

Fig. S20. Country-specific effects of different driving factors on the changes in DAPP from 2015 to 2030. Please refers to Fig. S1 for detailed definitions of ASIA, OECD, MAF, REF and LAM.

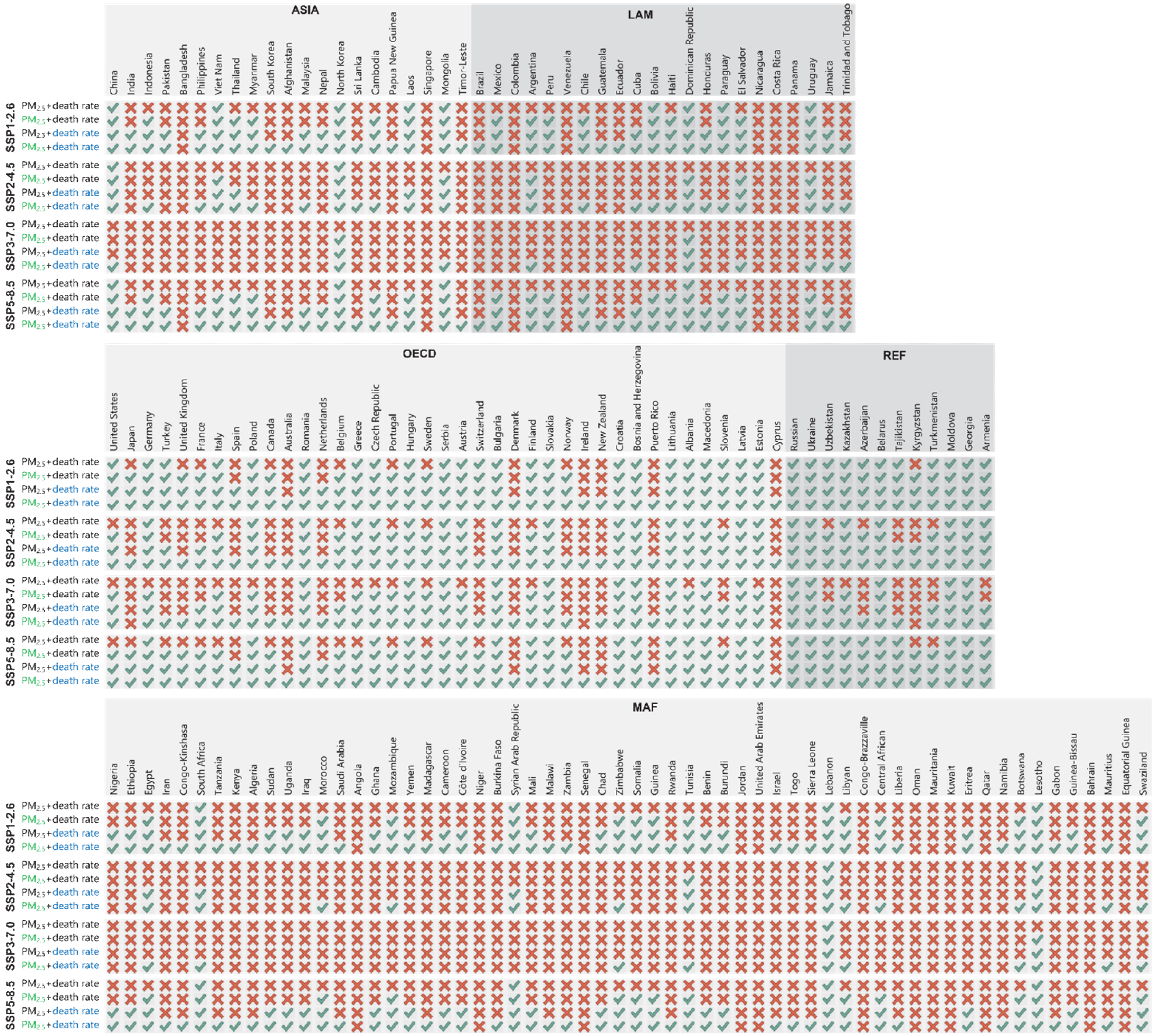

Fig. S21. Country-specific attainment of SDG3.9 by 2030 under different combinations of scenarios as well as additional improvement in air pollution control and healthcare. Colors in the legends indicate 20% lower $PM_{2.5}$ concentration (green) and death rate of diseases (blue) relative to the projected value because of additional improvement in air pollution control and healthcare. The green tick represents the attainment of moderate SDG3.9 target (20% lower than 2015). Please refers to Fig. S1 for detailed definitions of ASIA, OECD, MAF, REF and LAM.

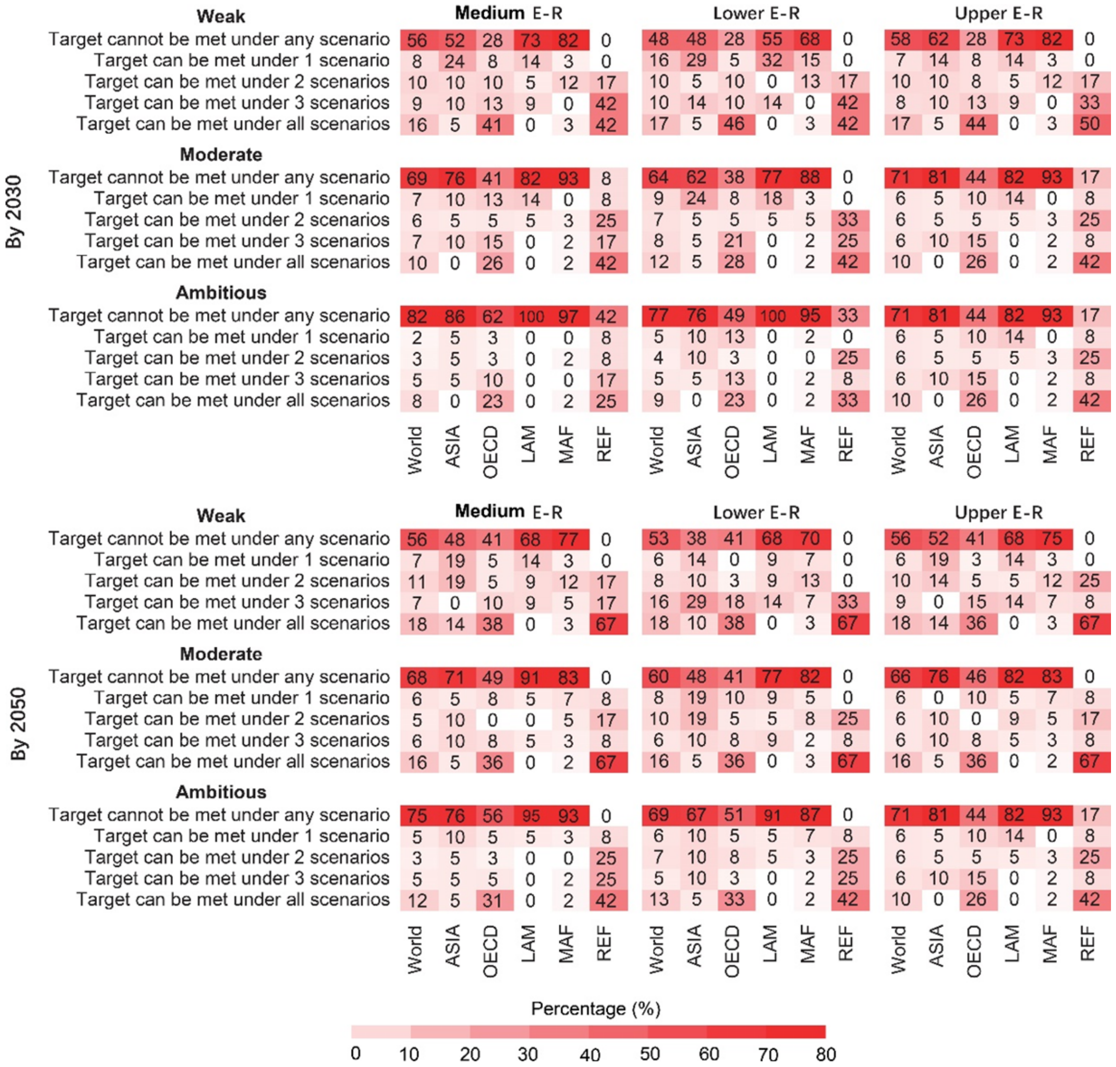

Fig. S22. Attainment of SDG3.9 based on the medium, lower and upper value of the exposure-response function (E-R). The colors indicate percentages of countries that can meet SDG3.9 under 0, 1, 3, 2, and 4 possible scenarios assessed with, weak, moderate and ambitious settings for SDG3.9 represented as a 10%, 20% and 30% reduction in DAPP relative to 2015, respectively. Please refers to Fig. S1 for detailed definitions of ASIA, OECD, MAF, REF and LAM.

Table S1. Comparative summary of scenario, input data and major results among previous studies.

Reference	Source	Study area	$PM_{2.5}$ concentration projection		Socioeconomic factors	Changes in DAPP (deaths attributable to $PM_{2.5}$ pollution)
			Model	Scenario		
Huang et al. (2023)[30]	Nature Sustainability	World	TM5-FASST	30000 state of worlds sampled by considering five set of SSPs and canbon price.	Population, age structure and baseline death rate in line with SSPs.	With pricing carbon, ensemble-median DAPP decreased 0.5 million annually from 2015 to 2100 on average.
Cheng et al (2023)[31]	One Earth	China	WRF-CMAQ	Specific scenarios defined by clean air and carbon neutrality policy.	Population, age structure in line with SSP1 and baseline death rate change by time.	DAPP is expected to decrease from 1.36 in 2020 to 1.28 in 2030 via the combination of carbon-peak, carbon-neutrality, and air pollution control policies
Yang et al. (2022)[26]	Nature Sustainability	World	GFDL-ESM4.1	SSP1-1.9, SSP1-2.6, SSP2-4.5, SSP3-7.0 and SSP5-8.5	Popluation, age structure and baseline death rate in line with SSPs.	DAPP is expected to increase by 7.3% (SSP1-1.9) -54.9% (SSP5-8.5) from 2015 to 2030.
Sliva et al. (2017)[18]	Nature Climate Change	World	5 models from CMIP5	RCP8.5, assuming the emissions fixed	Population, age structure and baseline death rate change by time.	DAPP is expected to increase by 3.3% from 2000 to 2030.
Hong et al. (2019)[27]	Proceedings of the National Academy of Sciences	China	WRF-CMAQ	RCP4.5, assuming the emissions fixed	Population and baseline death rate change by time while assuming fixed age structure.	DAPP is expected to increase by 27 thousand from 2006-2010 to 2046-2050.
Markandya et al. (2009)[28]	Lancet	Europe, India and China	TM5-FASST	Business as usual scenario, assuming the meteroloigical field fixed	Baseline death rate change over time, did not differentiate by age structure.	DAPP per million people is expected to decrease by 41% and 4% in Europe and China, while increasing by 38% in India from 2010 to 2030.
Lelieveld et al. (2015)[14]	Nature	World	ECHAM5	Business as usual scenario, assuming the meterological field fixed	Population and age structure change over time while assuming fixed baseline death rate.	DAPP is expected to increase by 97% from 2010 to 2050.
Fang et al. (2013)[29]	Climatic Change	World	GFDL-AM3	SRES-A1B	Population, age structure and baseline death rate fixed.	DAPP is expected to increase by 4.4% from 2000 to 2090.
Rafaj et al. (2018)[30]	Global Environmental Change	World	EMEP MSC-W	3 energy-related scenarios based on World Energy Model, assuming the meteroloigical field fixed	Population and age atructure change over time while assuming contributions from individual diseases to total deaths within each age group remain constant.	DAPP is expected to increase by 40% from 2015 to 2040, under the scenario assuming the continuation of existing and planned policies.
West et al. (2013)[31]	Nature Climate Change	World	MOZART-4	RCP4.5	Population and baseline death rate change over time, did not differentiate age structure.	DAPP is expected to increase by 0.5 million in 2030, but decrease by 2.4 million in 2050 relative to 2000.
Sliva et al. (2016)[32]	Atmospheric Chemistry and Physics	World	6 models from CMIP5	RCP2.6, RCP4.5, RCP6.0, and RCP8.5	Population, age structure and baseline death rate change over time.	DAPP is expected to increase from 1.7 million in 2000 to 2.5 million in 2030, and then drop to 1.8 million in 2050 under RCP4.5.
Chowdhury et al. (2018)[19]	Nature Communications	India	13 models from CMIP5	RCP4.5 and RCP8.5	Population, age structure and baseline death rate change by time.	DAPP is expected to decrease by 12% (RCP4.5) from 2011-2020 to 2031-2040.
Yang et al. (2019)[33]	Environmental Research	United States	WRF-Chem	RCP4.5 and RCP8.5	Population and baseline death rate change over time, did not differentiate age structure.	DAPP is expected to decrease by 63 thousand (RCP4.5) and 82 thousand (RCP8.5) in 2030, relative to 2005.

Table S2. Number of countries that achieved the moderate SDG3.9 by 2030 under different scenarios.

	Number of attainable scenarios	SSP1-2.6	SSP2-4.5	SSP3-7.0	SSP5-8.5	World	ASIA	OECD	LAM	MAF	REF
By 2030	0					107	16	16	18	56	1
	1	√				11	2	5	3		1
			√								
				√							
					√						
	2	√	√			1		1			
		√		√							
		√			√	8	1	1	1	2	3
			√	√							
			√		√						
				√	√						
	3	√	√	√							
		√		√	√						
		√	√		√	11	2	6		1	2
			√	√	√						
	4	√	√	√	√	16		1		1	5

Note: Please refer to Fig. S1 for detailed definitions of ASIA, OECD, MAF, REF and LAM.

Table S3. Details of input data for $PM_{2.5}$ concentration projection.

Model	Scenario	Ensembles				
INM-CM4-8	SSP1-2.6	r1i1p1f1				
	SSP2-4.5	r1i1p1f1				
	SSP3-7.0	r1i1p1f1				
	SSP5-8.5	r1i1p1f1				
INM-CM5-0	SSP1-2.6	r1i1p1f1				
	SSP2-4.5	r1i1p1f1				
	SSP3-7.0	r1i1p1f1	r2i1p1f1	r3i1p1f1	r4i1p1f1	r5i1p1f1
	SSP5-8.5	r1i1p1f1				
GISS-E2-1-G	SSP1-2.6	r1i1p3f1				
	SSP2-4.5	r1i1p3f1	r2i1p3f1	r3i1p3f1	r4i1p3f1	r5i1p3f1
	SSP3-7.0	r1i1p3f1				
	SSP5-8.5	r1i1p3f1				
GFDL-ESM4	SSP1-2.6	r1i1p1f1				
	SSP2-4.5	r1i1p1f1				
	SSP3-7.0	r1i1p1f1				
	SSP5-8.5	r1i1p1f1				
MRI-ESM2-0	SSP1-2.6	r1i1p1f1				
	SSP2-4.5	r1i1p1f1	r2i1p1f1			
	SSP3-7.0	r1i1p1f1	r2i1p1f1	r3i1p1f1	r4i1p1f1	r5i1p1f1
	SSP5-8.5	r1i1p1f1	r1i2p1f1			
MIROC-ES2L	SSP1-2.6	r1i1p1f2	r2i1p1f2	r3i1p1f2		
	SSP2-4.5	r1i1p1f2				
	SSP3-7.0	r1i1p1f2				
	SSP5-8.5	r1i1p1f2				
NorESM2-LM	SSP1-2.6	r1i1p1f1				
	SSP2-4.5	r1i1p1f1	r2i1p1f1	r3i1p1f1		
	SSP3-7.0	r1i1p1f1	r2i1p1f1	r3i1p1f1		
	SSP5-8.5	r1i1p1f1				
NorESM2-MM	SSP1-2.6	r1i1p1f1				
	SSP2-4.5	r1i1p1f1				
	SSP3-7.0	r1i1p1f1				
	SSP5-8.5	r1i1p1f1				
GFDL-CM4	SSP1-2.6					
	SSP2-4.5	r1i1p1f1				
	SSP3-7.0					
	SSP5-8.5	r1i1p1f1				
CNRM-ESM2-1	SSP1-2.6					
	SSP2-4.5					
	SSP3-7.0	r1i1p1f2	r2i1p1f2	r3i1p1f2		
	SSP5-8.5					
BCC-ESM1	SSP1-2.6					
	SSP2-4.5					
	SSP3-7.0	r1i1p1f1	r2i1p1f1	r3i1p1f1		
	SSP5-8.5					

Note: The data was projected using several initial states, initialization methods or physics details. The name of ensembles indicates different settings in the realization (r), initialization (i), perturbation (p) and forcing (f), which represent the variability of simulation data concerning a single model.

References

1 International Institute for Applied Systems Analysis. *SSP Database*, <https://tntcat.iiasa.ac.at/SspDb/dsd?Action=htmlpage&page=10#v2> (2018).

2 Lutz, W., Anne, G., Samir, K., Marcin, S. & Nikolaos, S. *Demographic and Human Capital Scenarios for the 21st Century: 2018 assessment for 201 countries*, <http://www.who.int/phe/publications/air-pollution-global-assessment/en/> (2018).

3 Goldewijk, K., Beusen, A., Doelman, J. & Stehfest, E. New anthropogenic land use estimates for the Holocene; HYDE 3.2. *Earth System Science Data Discussions*, 1-40, doi:doi:10.5194/essd-2016-58, 2016 (2017).

4 Hammer, M. S. *et al.* Global Estimates and Long-Term Trends of Fine Particulate Matter Concentrations (1998–2018). *Environmental Science & Technology* **54**, 7879-7890, doi:10.1021/acs.est.0c01764 (2020).

5 Eyring, V. *et al.* Overview of the Coupled Model Intercomparison Project Phase 6 (CMIP6) experimental design and organization. *Geoscientific Model Development* **9**, 1937-1958, doi:10.5194/gmd-9-1937-2016 (2016).

6 GBD 2019 Diseases and Injuries Collaborators. Global burden of 369 diseases and injuries in 204 countries and territories, 1990–2019: a systematic analysis for the Global Burden of Disease Study 2019. *The Lancet* **396**, 1204-1222, doi:https://doi.org/10.1016/S0140-6736(20)30925-9 (2020).

7 World Bank. *GNI per capita, Atlas method (current US$)*, <https://data.worldbank.org/indicator/NY.GNP.PCAP.CD?view=chart> (2018).

8 Dellink, R., Chateau, J., Lanzi, E. & Magné, B. Long-term economic growth projections in the Shared Socioeconomic Pathways. *Global Environmental Change* **42**, 200-214, doi:10.1016/j.gloenvcha.2015.06.004 (2017).

9 Murray, C. J. L. & Lopez, A. D. On the Comparable Quantification of Health Risks: Lessons from the Global Burden of Disease Study. *Epidemiology* **10**, 594-605 (1999).

10 World Health Organization. *Ambient air pollution: A global assessment of exposure and burden of disease*, <http://www.who.int/phe/publications/air-pollution-global-assessment/en/> (2016).

11 GBD 2019 Risk Factors Collaborators. Global burden of 87 risk factors in 204 countries and territories, 1990–2019: a systematic analysis for the Global Burden of Disease Study 2019. *The Lancet* **396**, 1223-1249, doi:https://doi.org/10.1016/S0140-6736(20)30752-2 (2020).

12 Cohen, A. *et al.* Estimates and 25-year trends of the global burden of disease attributable to ambient air pollution: an analysis of data from the Global Burden of Diseases Study 2015. *The Lancet* **389**, 1907-1918, doi:10.1016/s0140-6736(17)30505-6 (2017).

13 Lim, S. S. *et al.* A comparative risk assessment of burden of disease and injury attributable to 67 risk factors and risk factor clusters in 21 regions, 1990-2010: a systematic analysis for the Global Burden of Disease Study 2010. *The Lancet* **380**, 2224-2260, doi:10.1016/S0140-6736(12)61766-8 (2012).

14 Lelieveld, J., Evans, J., Fnais, M., Giannadaki, D. & Pozzer, A. The contribution of outdoor air pollution sources to premature mortality on a global scale. *Nature* **525**, 367-371, doi:10.1038/nature15371 (2015).

15 Rockhill, B., Newman, B. & Weinberg, C. Use and misuse of population attributable fractions.

American Journal of Public Health **88**, 15-19, doi:https://doi.org/10.2105/ajph.88.1.15 (1998).
16 Burnett, R. *et al.* Global estimates of mortality associated with long-term exposure to outdoor fine particulate matter. *Proceedings of the National Academy of Sciences of the United States of America* **115**, 9592-9597, doi:10.1073/pnas.1803222115 (2018).
17 Burnett, R. & Cohen, A. Relative Risk Functions for Estimating Excess Mortality Attributable to Outdoor PM2.5 Air Pollution: Evolution and State-of-the-Art. *Atmosphere* **11**, 589, doi:10.3390/atmos11060589 (2020).
18 Silva, R. *et al.* Future global mortality from changes in air pollution attributable to climate change. *Nature Climate Change* **7**, 647-651, doi:10.1038/nclimate3354 (2017).
19 Rao, S. *et al.* Future air pollution in the Shared Socio-economic Pathways. *Global Environmental Change* **42**, 346-358, doi:https://doi.org/10.1016/j.gloenvcha.2016.05.012 (2017).
20 Gidden, M. J. *et al.* Global emissions pathways under different socioeconomic scenarios for use in CMIP6: a dataset of harmonized emissions trajectories through the end of the century. *Geoscientific Model Development* **2018**, 1-42, doi:10.5194/gmd-2018-266 (2018).
21 Turnock, S. T. *et al.* Historical and future changes in air pollutants from CMIP6 models. *Atmospheric Chemistry and Physics* **2020**, 1-40, doi:10.5194/acp-2019-1211 (2020).
22 Chowdhury, S., Dey, S. & Smith, K. R. Ambient PM2.5 exposure and expected premature mortality to 2100 in India under climate change scenarios. *Nature Communications* **9**, 318, doi:10.1038/s41467-017-02755-y (2018).
23 Silva, R. *et al.* Global premature mortality due to anthropogenic outdoor air pollution and the contribution of past climate change. *Environmental Research Letters* **8**, 034005, doi:10.1088/1748-9326/8/3/034005 (2013).
24 Silva, R. A. *et al.* The effect of future ambient air pollution on human premature mortality to 2100 using output from the ACCMIP model ensemble. *Atmospheric Chemistry and Physics* **16**, 9847-9862, doi:10.5194/acp-16-9847-2016 (2016).
25 Jerrett, M. *et al.* Comparing the Health Effects of Ambient Particulate Matter Estimated Using Ground-Based versus Remote Sensing Exposure Estimates. *Environmental Health Perspectives* **125**, 552-559, doi:10.1289/ehp575 (2017).
26 Foreman, K. J. *et al.* Forecasting life expectancy, years of life lost, and all-cause and cause-specific mortality for 250 causes of death: reference and alternative scenarios for 2016-40 for 195 countries and territories. *The Lancet* **392**, 2052-2090, doi:10.1016/s0140-6736(18)31694-5 (2018).
27 Girosi, F. & King, G. *Demographic Forecasting*, <https://gking.harvard.edu/node/5502> (2008).
28 National Platform for Common Geospatial Information Services of China. *World Map*, <https://www.tianditu.gov.cn/> (2023).
29 US National Geophysical Data Center. *ETOPO1, Global 1 Arc-minute Ocean Depth and Land Elevation*, <https://rda.ucar.edu/datasets/ds759.4/> (2009).
30 Huang, X., Srikrishnan, V., Lamontagne, J., Keller, K. & Peng, W. Effects of global climate mitigation on regional air quality and health. *Nature Sustainability*, doi:10.1038/s41893-023-01133-5 (2023).
31 Cheng, J. *et al.* A synergistic approach to air pollution control and carbon neutrality in China can avoid millions of premature deaths annually by 2060. *One Earth* **6**, 978-989,

doi:10.1016/j.oneear.2023.07.007 (2023).
