# Supplementary material for: Substantially reducing global PM2.5-related deaths under SDG3.9 requires better air pollution control and healthcare
Source: Nat Commun. 2024 Mar 28;15:2729. doi: 10.1038/s41467-024-46969-3 (PMC10978932; doi:10.1038/s41467-024-46969-3)
Supplement: Supplementary file 3 — Reporting Summary [file 41467_2024_46969_MOESM3_ESM.pdf]

nature portfolio

Corresponding author(s): Chunyang He

Last updated by author(s): Feb 16, 2024

Reporting Summary

Nature Portfolio wishes to improve the reproducibility of the work that we publish. This form provides structure for consistency and transparency in reporting. For further information on Nature Portfolio policies, see our Editorial Policies and the Editorial Policy Checklist.

Statistics

For all statistical analyses, confirm that the following items are present in the figure legend, table legend, main text, or Methods section.

n/a	Confirmed	
☐	☒	The exact sample size (*n*) for each experimental group/condition, given as a discrete number and unit of measurement
☒	☐	A statement on whether measurements were taken from distinct samples or whether the same sample was measured repeatedly
☒	☐	The statistical test(s) used AND whether they are one- or two-sided<br>*Only common tests should be described solely by name; describe more complex techniques in the Methods section.*
☒	☐	A description of all covariates tested
☒	☐	A description of any assumptions or corrections, such as tests of normality and adjustment for multiple comparisons
☐	☒	A full description of the statistical parameters including central tendency (e.g. means) or other basic estimates (e.g. regression coefficient) AND variation (e.g. standard deviation) or associated estimates of uncertainty (e.g. confidence intervals)
☒	☐	For null hypothesis testing, the test statistic (e.g. *F*, *t*, *r*) with confidence intervals, effect sizes, degrees of freedom and *P* value noted<br>*Give P values as exact values whenever suitable.*
☒	☐	For Bayesian analysis, information on the choice of priors and Markov chain Monte Carlo settings
☒	☐	For hierarchical and complex designs, identification of the appropriate level for tests and full reporting of outcomes
☒	☐	Estimates of effect sizes (e.g. Cohen's *d*, Pearson's *r*), indicating how they were calculated

Our web collection on statistics for biologists contains articles on many of the points above.

Software and code

Policy information about availability of computer code

Data collection	No code or software was used in data collection.
Data analysis	We used the integrated exposure-response function updated in the Global Burden of Disease 2019 to estimate the relative risk caused by PM2.5 exposure. The detailed function is accessible to all users at https://ghdx.healthdata.org/record/ihme-data/gbd-2019-burden-by-risk-1990-2019. Custom Python (3.8.3) scripts for estimating disease mortality and the functions used for estimating the deaths attributable to PM2.5 air pollution embedded in the Microsoft Excel file were available at Zenodo (doi: 10.5281/zenodo.8128795). Other data processing and map creation in this study are conducted at ArcGIS (10.6) and Microsoft Excel (16.74).

For manuscripts utilizing custom algorithms or software that are central to the research but not yet described in published literature, software must be made available to editors and reviewers. We strongly encourage code deposition in a community repository (e.g. GitHub). See the Nature Portfolio guidelines for submitting code & software for further information.

Data

Policy information about availability of data

All manuscripts must include a data availability statement. This statement should provide the following information, where applicable:

- Accession codes, unique identifiers, or web links for publicly available datasets
- A description of any restrictions on data availability
- For clinical datasets or third party data, please ensure that the statement adheres to our policy

All the data presented in the main text are openly available at the Github repository https://github.com/yuehuanbi/attainment-of-SDG3.9. Historical population data was derived from the World Population Prospects 2019 (https://population.un.org/wpp/). The future projection of population and age structure was derived from the Wittgenstein Centre Human Capital Data (WIC2.0) (http://dataexplorer.wittgensteincentre.org/wcde-v2/) developed by IIASA. The gridded population data were obtained from the History Database of the Global Environment (HYDE3.2) published by the Netherlands Environmental Assessment Agency (ftp://ftp.pbl.nl/hyde). Historical estimation of PM2.5 concentrations were obtained from the Global Estimates of Fine Particulate Matter dataset (V4.GL.02) published by the Dalhousie University's Atmospheric Composition Analysis Group (http://fizz.phys.dal.ca/~atmos/). The future concentration of pollutants was derived from the Coupled Model Intercomparison Project Phase 6 (CMIP6) (https://pcmdi.llnl.gov/CMIP6/). Historical death rate of diseases was accessed from the Global Burden of Disease (GBD) 2019 (http://ghdx.healthdata.org/gbd-results-tool). The fertility and mean years of education data was derived from the WIC2.0 dataset (http://dataexplorer.wittgensteincentre.org/wcde-v2/). Historical GDP per capita was sourced from publicly available World Bank data and future GDP per capita data was sourced from the projections conducted by the OECD contained in the SSPs database (https://tntcat.iiasa.ac.at/SspDb/).

Research involving human participants, their data, or biological material

Policy information about studies with human participants or human data. See also policy information about sex, gender (identity/presentation), and sexual orientation and race, ethnicity and racism.

Reporting on sex and gender	This study doesn't involve sex or gender.
Reporting on race, ethnicity, or other socially relevant groupings	This is not applicable in this study.
Population characteristics	This is not applicable in this study.
Recruitment	This is not applicable in this study.
Ethics oversight	This is not applicable in this study.

Note that full information on the approval of the study protocol must also be provided in the manuscript.

Field-specific reporting

Please select the one below that is the best fit for your research. If you are not sure, read the appropriate sections before making your selection.

☐ Life sciences ☐ Behavioural & social sciences ☒ Ecological, evolutionary & environmental sciences

For a reference copy of the document with all sections, see nature.com/documents/nr-reporting-summary-flat.pdf

Ecological, evolutionary & environmental sciences study design

All studies must disclose on these points even when the disclosure is negative.

Study description	We estimated the annual deaths attributable to ambient PM2.5 pollution from 2015 to 2050 at a pixel scale (10km) by combining an epidemiological model and climate scenarios.
Research sample	We used age- and disease specific death rate data from Global Burden of Disease 2019 (https://vizhub.healthdata.org/gbd-results/). Six kinds of diseases related to PM2.5 pollution were considered in this study, including lung cancer, chronic obstructive pulmonary disease, lower respiratory infection, ischemic heart disease, stroke, and diabetes mellitus type 2. Fifteen age groups were included in the equation, i.e., 25–30, 30–35...90–95, and beyond 95 years old. Although PM2.5 pollution is also related to other adverse birth outcomes including low birth weight and short gestation, these 6 diseases represent around 95% of total deaths related to PM2.5 pollution from all causes based on the estimation of GBD 2019. We did not consider differences by gender to reduce the complexity of our estimation.
Sampling strategy	The 5-year age group and the 6 selected diseases were chosen following the Global Burden of Disease 2019 study (https://vizhub.healthdata.org/gbd-results/). Evidence linking these diseases with exposure to ambient air pollution was judged to be consistent with a causal relationship on the basis of criteria specified for Global Burden of Disease (GBD) risk factors, including meta-analysis, cohort study, and biologically plausible relationship. Such strategy is widely adopted in related studies.

Data collection	The PM2.5 data, demographic data and death rate data were download directly. Historical population data was derived from the World Population Prospects 2019 (https://population.un.org/wpp/). The future projection of population and age structure was derived from the Wittgenstein Centre Human Capital Data (WIC2.0) (http://dataexplorer.wittgensteincentre.org/wcde-v2/) developed by IIASA. The gridded population data were obtained from the History Database of the Global Environment (HYDE3.2) published by the Netherlands Environmental Assessment Agency (ftp://ftp.pbl.nl/hyde). Historical estimation of PM2.5 concentrations were obtained from the Global Estimates of Fine Particulate Matter dataset (V4.GL.02) published by the Dalhousie University's Atmospheric Composition Analysis Group (http://fizz.phys.dal.ca/~atmos/). The future concentration of pollutants was derived from the Coupled Model Intercomparison Project Phase 6 (CMIP6) (https://pcmdi.llnl.gov/CMIP6/). Historical death rate of diseases was accessed from the Global Burden of Disease (GBD) 2019 (http://ghdx.healthdata.org/gbd-results-tool). The fertility and mean years of education data was derived from the WIC2.0 dataset (http://dataexplorer.wittgensteincentre.org/wcde-v2/). Historical GDP per capita was sourced from publicly available World Bank data and future GDP per capita data was sourced from the projections conducted by the OECD contained in the SSPs database (https://tntcat.iiasa.ac.at/SspDb/).
Timing and spatial scale	The data used in this study were collected between January 2021 to April 2023. The PM2.5 data have a resolution of 0.01 degrees. Population distribution data have a spatial resolution of 0.083 degrees. Data on the age structure, as well as the age- and disease-specific death rates were obtained from the United Nations and Global Burden of Disease 2019 dataset, at a national scale. During the historical period, all these data a available annually from 2000 to 2015, the future projection include data in 2030 and 2050.
Data exclusions	No data were excluded in this study.
Reproducibility	Three attempts to repeat the results, which were independently conducted by our co-authors, were successful.
Randomization	N/A. This study does not involve group allocation and sample randomization.
Blinding	N/A. This study does not involve group allocation and sample randomization.

Did the study involve field work? ☐ Yes ☒ No

Reporting for specific materials, systems and methods

We require information from authors about some types of materials, experimental systems and methods used in many studies. Here, indicate whether each material, system or method listed is relevant to your study. If you are not sure if a list item applies to your research, read the appropriate section before selecting a response.

Materials & experimental systems

n/a	Involved in the study
☒	☐ Antibodies
☒	☐ Eukaryotic cell lines
☒	☐ Palaeontology and archaeology
☒	☐ Animals and other organisms
☒	☐ Clinical data
☒	☐ Dual use research of concern
☒	☐ Plants

Methods

n/a	Involved in the study
☒	☐ ChIP-seq
☒	☐ Flow cytometry
☒	☐ MRI-based neuroimaging

Plants

Seed stocks	N/A. This study does not involve group allocation and sample randomization.
Novel plant genotypes	N/A. This study does not involve group allocation and sample randomization.
Authentication	N/A. This study does not involve group allocation and sample randomization.
